# Symmetry-protected topological order in a 100-site spin chain on a digital quantum computer

**DOI:** 10.1038/s41534-026-01334-8

**Published:** 2026-09-01

**Authors:** George Pennington, Kevin C. Smith, James R. Garrison, Lachlan P. Lindoy, Jason Crain, Ben Jaderberg

**Affiliations:** 1 The Hartree Centre, STFC, Sci-Tech Daresbury, Warrington, UK; 2 IBM Quantum, IBM Research Cambridge, Cambridge, MA USA; 3 IBM Quantum, Thomas J. Watson Research Centre, Yorktown Heights, NY USA; 4 National Physical Laboratory, Teddington, UK; 5 IBM Research Europe, The Hartree Centre, Warrington, UK; 6 Department of Physics, Clarendon Laboratory, University of Oxford, Oxford, UK; 7 IBM Quantum, IBM Research Europe, Winchester, UK

**Keywords:** Mathematics and computing, Physics

## Abstract

Symmetry-protected topological (SPT) systems extend the Landau paradigm of quantum matter by admitting distinct phases that lack local order parameters, making them challenging to characterise using conventional experimental probes. While material platforms provide important evidence for SPT order through indicative signatures such as boundary states, experimental access is typically limited to such partial probes rather than fully diagnostic measurements of the underlying quantum state. Programmable quantum processors offer a powerful complementary approach, enabling direct access to non-local properties of many-body wavefunctions. However, preparing such states on digital hardware at scales sufficient to avoid finite-size effects is typically limited by the circuit depth required to capture non-trivial entanglement. Here, we use a tensor-network-based approximate quantum compiling (AQC) protocol to construct shallow quantum circuits (18–39 CNOT depth) that prepare 100-site ground states of the spin-1/2 bond-alternating Heisenberg chain across distinct SPT phases with 97.9–99.0% fidelity. Executing these circuits on IBM quantum hardware, we directly extract multiple non-local diagnostics of SPT order, including string order for lengths up to 20, persisting well beyond the decay of conventional two-point correlations, characteristic features of the entanglement spectrum, and symmetry-protected edge modes. The simultaneous observation of these independent diagnostics provides a scalable and programmable approach to preparing and characterising SPT phases on quantum processors. More broadly, this establishes digital quantum devices as flexible platforms for studying complex quantum matter and provides a practical foundation for exploring non-equilibrium dynamics in regimes that challenge classical computational methods.

## Introduction

The discovery of symmetry-protected topological (SPT) phases has transformed our understanding of quantum matter by revealing classifications of order that transcend the Landau paradigm^1^. Unlike conventional phases characterised by local order parameters, SPT phases preserve the protecting symmetries and are diagnosed by non-local or entanglement-based invariants together with symmetry-protected edge states that are robust to symmetry-preserving perturbations^2,3^. As a result, SPT phases have become a central topic in modern condensed matter physics^1^. These features not only enrich the taxonomy of quantum phases, but also underpin emerging applications in quantum information science^4^, including topologically protected spin transport^5^, low-dissipation interconnects^6^ and robust memory elements^7^.

SPT physics has been explored experimentally in a number of platforms including cold atoms^8,9^, Rydberg analogue simulators^10,11^, trapped-ion systems^12^, and two material realisations of the Haldane phase using nanographene, namely: the spin-1/2 bond-alternating Heisenberg chain (BAHC)^13^ and the spin-1 bilinear-biquadratic (BLQP) model^14^. The latter material realisations of SPT order provide elegant physical instances of these phases. While such solid-state platforms realise the target physics, they face two important limitations. First, they exhibit structural inflexibility: the bond alternation and boundary conditions are fixed during synthesis by the chemical linkages of the molecular precursors, limiting the ability to tune Hamiltonian parameters in situ. Second, the nature of available experimental probes restricts the characterisation of the resulting SPT phase. Diagnosing SPT order in these systems typically relies on indirect, boundary-restricted signatures, such as the spectroscopic detection of fractionalised edge excitations or the mapping of bulk excitations using scanning tunnelling microscopy (STM), leaving direct access to the underlying bulk wave function severely limited.

Programmable digital quantum processors offer a powerful complementary approach, bridging the gap between physical realisation and microscopic observability. In this setting, the SPT state is physically realised on the quantum hardware through circuit-based state preparation. Crucially, the programmable nature of the platform addresses both limitations of static material systems: site-resolved control enables the tuning of system size, Hamiltonian parameters, and boundary conditions within a single device, while measurement-basis rotations offer a route to direct probes of the bulk wave function. This enables the extraction of a wide range of non-local observables, including string order across extended distances and characteristic entanglement degeneracies across multiple spatial cuts. Digital quantum architectures therefore provide a highly flexible and programmable platform for studying complex quantum matter, with access to microscopic observables that are difficult to obtain in conventional experimental systems. Furthermore, going beyond static properties, digital quantum processors offer the prospect of studying dynamical properties of SPT systems, under arbitrary quench Hamiltonians.

Recent work has begun to demonstrate the promise of digital quantum platforms toward probing nontrivial SPT physics. For the canonical example of the spin-1 Affleck-Kennedy-Lieb-Tasaki (AKLT) state^15^, methodological innovations have enabled efficient preparation of small systems using digital quantum computers^16–18^. Ground state preparation in the SPT phase of the cluster-Ising model has also seen steady progress^19–22^ including for systems as large as 80 sites^23^. Yet preparing ground states beyond minimally-entangled examples, whilst maintaining large system sizes and verifying their preparation to high fidelity, remains an open problem. In particular, the ability to study spin-1/2 models with only two-body exchange interactions would bring digital quantum simulation closer to the microscopic Hamiltonians recently realised in engineered spin materials^13^.

In this work, we make major strides toward addressing these challenges by preparing 100-site ground states of both SPT phases of the spin-1/2 BAHC on IBM superconducting quantum computers. We verify our preparation through observation of non-local string order for strings of up to length 20, detection of edge modes, and qualitative observation of signature entanglement spectrum degeneracies. We achieve this by applying an approximate quantum compiling (AQC) protocol to obtain shallow quantum circuit representations of matrix product states (MPSs) produced by the density matrix renormalisation group (DMRG) algorithm. For four points in the phase diagram, we obtain ground states with up to bond dimension *χ* = 40, and construct circuits of up to 39 CNOT depth whilst maintaining circuit fidelities of at least 97.9% with the DMRG ground states. This enables a major step forward in the preparation of SPT states on quantum hardware. For example, prior work demonstrated the preparation of 80-site MPSs^23^, yet was limited to just bond dimension 2. Using our compiled circuits, we observe non-local string order across up to 20 sites, evidence of characteristic degeneracies in the entanglement spectrum, and experimental observation of the symmetry-protected edge states, thereby verifying preparation through signature properties of the SPT order on quantum hardware. Our method establishes a practical foundation for further research requiring high-fidelity ground state preparation, such as the simulation of non-equilibrium SPT dynamics^24–26^, or the use of SPT states as a resource for measurement-based quantum computation^27^.

**Fig. 1:**
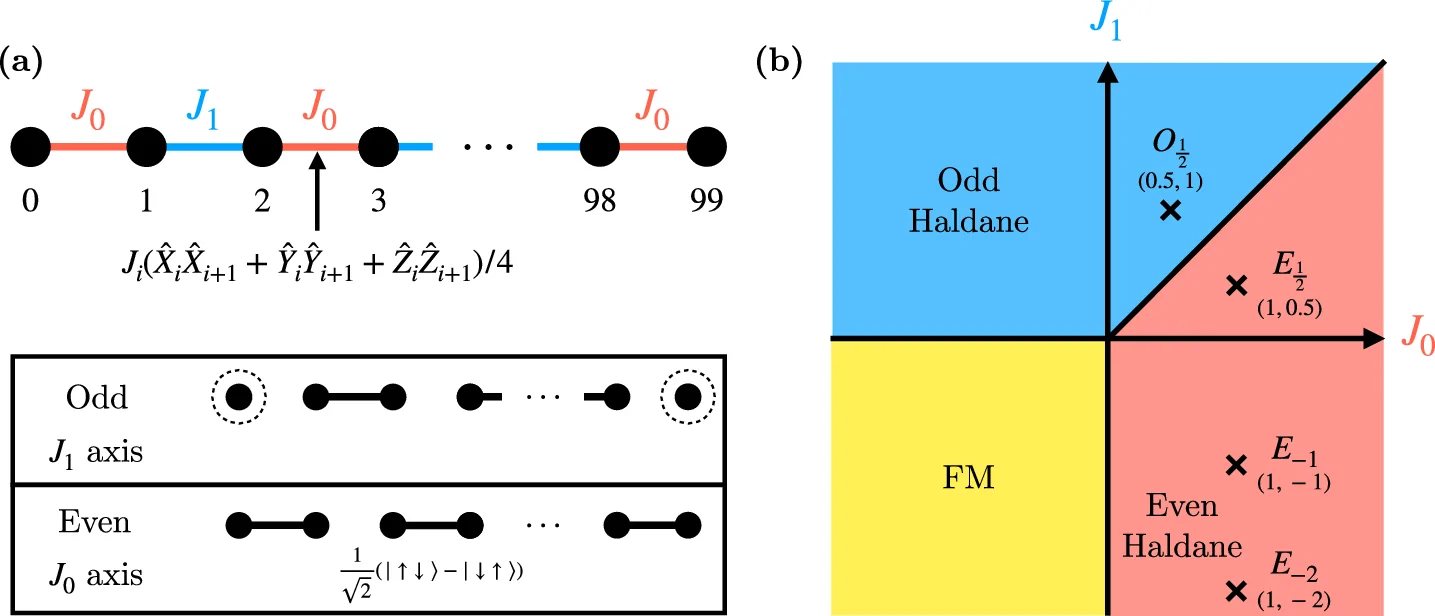
The 100-site spin-1/2 bond-alternating Heisenberg chain. **a** (Top) A diagram representing the model with alternating *J*_0_, *J*_1_ couplings. (Bottom) a schematic representation of the ground states for the positive *J*_0_ and *J*_1_ axes, consisting of simple products of singlet pairs: $1/\sqrt{2}(\left\vert \uparrow \downarrow \right\rangle -\left\vert \downarrow \uparrow \right\rangle )$, plus decoupled edge spins in the odd-Haldane phase. **b** The phase diagram of the model^28,29^. The model exhibits two symmetry-protected topological phases: the odd-Haldane phase for *J*_1_ > 0 and *J*_1_ > *J*_0_ and the even-Haldane phase for *J*_0_ > 0 and *J*_0_ > *J*_1_, along with a trivial ferromagnetic (FM) phase for *J*_0_ < 0 and *J*_1_ < 0. The black crosses denote the four points in the phase diagram considered in this work, which we denote as ${O}_{\frac{1}{2}}$, ${E}_{\frac{1}{2}}$, *E*_−1_, and *E*_−2_.

We consider the 1D spin-1/2 BAHC, defined by the Hamiltonian:

1 $$\hat{H}=\frac{1}{4}\mathop{\sum }\limits_{i=0}^{N-2}{J}_{i}\left({\hat{X}}_{i}{\hat{X}}_{i+1}+{\hat{Y}}_{i}{\hat{Y}}_{i+1}+{\hat{Z}}_{i}{\hat{Z}}_{i+1}\right),$$

with *J*_*i*_ = *J*_0_ if *i* is even, and *J*_*i*_ = *J*_1_ otherwise, and *N* = 100. Here $\hat{X}$, $\hat{Y}$, and $\hat{Z}$ are the Pauli operators. The model is characterised by alternating nearest-neighbour magnetic coupling strengths *J*_0_ and *J*_1_ as depicted in Fig. 1a. The phase diagram^28^ of this model is shown schematically in Fig. 1b, with the black crosses corresponding to the ground states considered in this work. There is a topologically-trivial ferromagnetic phase where both couplings are negative, as well as two phases exhibiting non-trivial SPT order, known as the odd-Haldane and even-Haldane phases. We consider four ground states in this work: three in the even-Haldane phase, with *J*_0_ = 1 and *J*_1_ = 0.5, − 1, − 2, and one in the odd-Haldane phase, with *J*_0_ = 0.5, *J*_1_ = 1. We note that, since *J*_0_ > 0 in the even-Haldane phase and *J*_1_ > 0 in the odd-Haldane phase, and the ground state is unchanged by a global rescaling of the Hamiltonian, we can identify each ground state by the ratio of the couplings, and label them as ${E}_{{J}_{1}/{J}_{0}}$ in the even-Haldane phase and ${O}_{{J}_{0}/{J}_{1}}$ in the odd-Haldane phase. Using this notation, we refer to the ground states considered in this work as ${O}_{\frac{1}{2}}$, ${E}_{\frac{1}{2}}$, *E*_−1_ and *E*_−2_. Along the positive *J*_0_ and *J*_1_ axes, the ground states are simple products of singlet pairs, with free, uncoupled, edge spins in the odd-Haldane phase (see Fig. 1a). It is interesting to note that the ${O}_{\frac{1}{2}}$ ground state considered in this work is similar to the state experimentally realised in nanographene in ref. ^13^, with *N* = 22 sites and the parametrisation *J*_0_/*J*_1_ ≈ 0.605. Thus, realising this model on quantum hardware is directly relevant to experimentally realisable materials.

Unlike the ferromagnetic phase, which is characterised by a local order parameter (magnetisation), the SPT phases are characterised by two non-local order parameters. In these phases, there is no conventional magnetic order: the single-site magnetisation, $\langle {\hat{Z}}_{i}\rangle$, is zero in the bulk, and standard two-point correlations, $\langle {\hat{Z}}_{i}{\hat{Z}}_{j}\rangle$, vanish exponentially with increasing separation. However, there is a form of hidden antiferromagnetic order, which is exposed by the non-local string order parameters. These are defined as the limit of expectation values of even-length Pauli strings^29^:

2 $${S}^{{\rm{E}}/{\rm{O}}}={\mathop{\lim }\limits_{l\to \infty }}\left(-\left\langle {\hat{Z}}_{s}{\rm{exp}}\left(\mathop{\sum }\limits_{i=s+1}^{s+l-2}\frac{i\pi }{2}{\hat{Z}}_{i}\right){\hat{Z}}_{s+l-1}\right\rangle \right),$$

where *s* denotes the left-most index of each string, and is even (odd) for the even (odd) string order parameter. *l* corresponds to the string length, and is even for both parameters. Intuitively, the string order measures the correlations between two sites, with the addition of a non-local phase dependent on the sites in between. Whilst conventional correlations vanish exponentially, the even (odd) string order parameter converges to a non-zero value in the even (odd) Haldane phase. For ease of notation and nomenclature, we refer to each length-*l* observable in the sequence in Eq. (2):

3 $${S}_{l,s}^{{\rm{E}}/{\rm{O}}}={(-1)}^{l/2}\left\langle \mathop{\bigotimes }\limits_{i=s}^{s+l-1}{\hat{Z}}_{i}\right\rangle ,$$

as the length-*l* string order parameter. Since the length-*l* string order rapidly tends toward its limiting value at a rate set by the correlation length (here only a few sites), it provides an excellent proxy for the true, infinite-length string order.

In addition to the string order parameters, a stronger indicator for the presence of SPT order can be found in the entanglement spectrum of a state^30^. The entanglement spectrum consists of the eigenvalues of the reduced density matrix of a bipartition of the chain, and contains information about how the two parts of the bipartition are entangled with each other. If the eigenvalues in the entanglement spectrum all have even degeneracy, then the entanglement across the bipartition cannot be adiabatically removed without either crossing a phase transition or breaking the symmetry of the system. On the other hand, if the eigenvalues all have odd degeneracy, the entanglement can be adiabatically removed. As detailed in ref. ^28^, the entanglement spectrum arising from a bipartition of the chain formed by cutting one of the *J*_0_ (*J*_1_) bonds exhibits eigenvalues with even (odd) degeneracy in the even-Haldane phase, signifying that the entanglement across *J*_0_ bonds is protected by the symmetry, whereas the entanglement across the *J*_1_ bonds is not. This is best understood by considering the case where the *J*_1_ coupling is adiabatically weakened to zero, in which case the ground state becomes a product of maximally-entangled singlet pairs coupled by the *J*_0_ bonds. In the odd-Haldane phase, the entanglement spectra exhibit a complementary pattern, where this time the *J*_1_ (*J*_0_) bonds exhibit even (odd) degeneracy. The alternating odd/even degeneracy in the entanglement spectrum is the most robust fingerprint of the SPT order. Whilst the above discussion strictly applies to infinite chains, in the finite chain case, a similar pattern should be observed when considering the entanglement spectra of *l*-site segments from the end of the chain, where the degeneracies should tend towards those of the infinite chain case, over a length scale comparable to the correlation length^16^.

Finally, since we consider the BAHC with *J*_0_ terminations, the entanglement across the bonds at either end of the chain can be adiabatically removed in the odd-Haldane phase, resulting in a decoupled free spin at each end of the chain when *J*_0_ = 0. In the case where *J*_0_ ≠ 0, we expect to see the free spin confined to the edge with a finite correlation length. In the even-Haldane phase, the entanglement across the *J*_0_ bonds cannot be adiabatically removed without breaking the symmetry, and thus in this phase, with these terminations, there is not a free edge spin.

## Results

### Ground state preparation

**Fig. 2:**
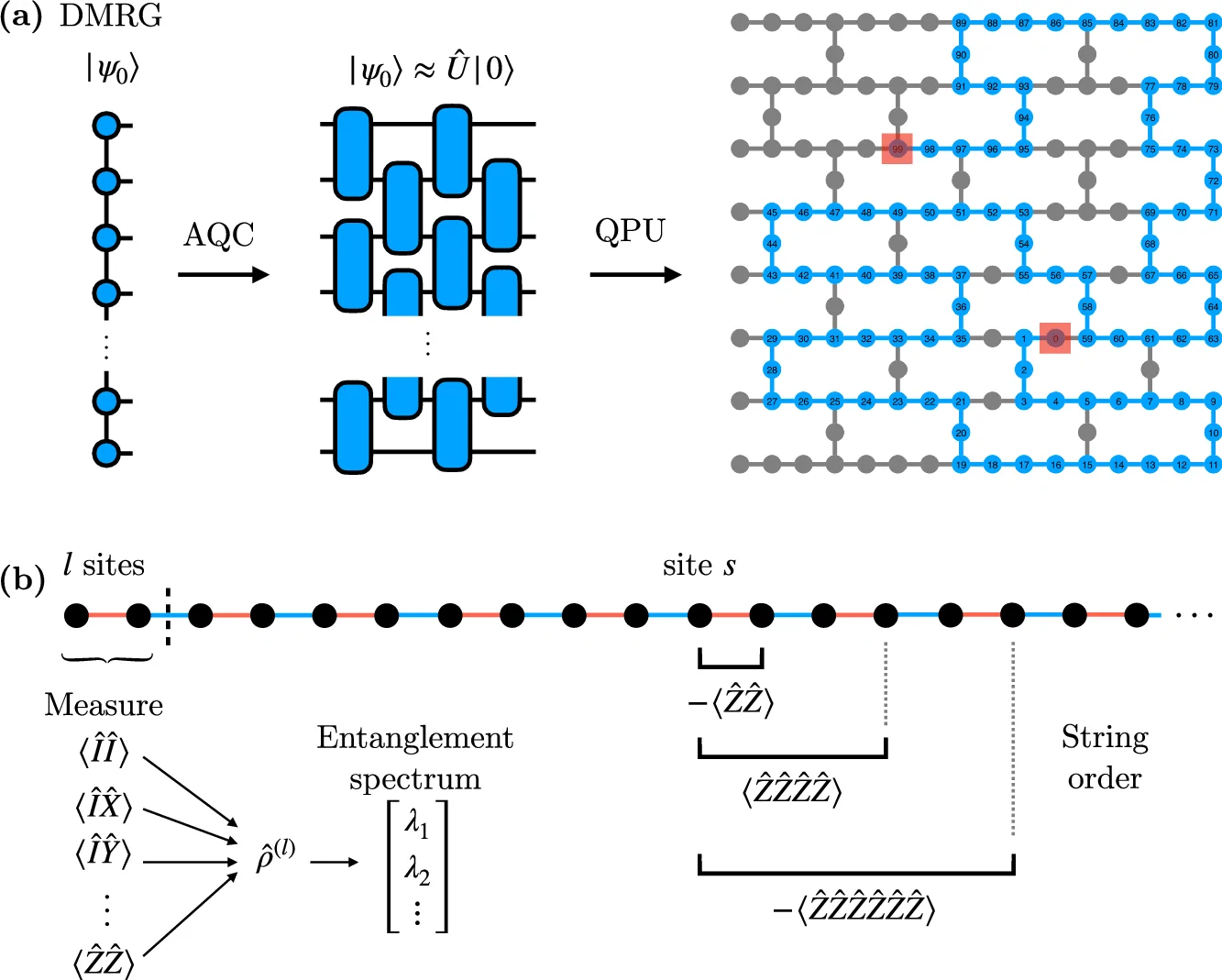
A schematic of our workflow. **a** We use the density matrix renormalisation group (DMRG) algorithm to find the ground state of the Hamiltonian, Eq. (1), as a matrix product state (MPS). Then we use approximate quantum compilation (AQC), specifically the AQC-Tensor^33^ algorithm with a brickwork ansatz, to approximately map the target MPS to a shallow quantum circuit. Finally, we execute the circuit on quantum hardware. The coupling map shown is that of the ibm_pittsburgh quantum processing unit (QPU), with the blue qubits and connections signifying those used for the experiment shown in Fig. 3, and the ends of the chain highlighted in red. **b** A sketch illustrating how the string order, Eq. (2), is measured (right), and the tomography procedure for obtaining the entanglement spectrum (left). Here the *λ*_*i*_'s are the sorted eigenvalues of the *l*-site reduced density matrix, ${\hat{\rho }}^{(l)}$, with *λ*_1_ ≥ *λ*_2_ ≥ . . . .

Figure 2 a gives an overview of the routine used to prepare the ground states on quantum hardware. We first obtain the MPS representation of each of the four 100-site ground states using the DMRG algorithm as implemented in the TeNPy library^31^. The resulting MPSs have bond dimensions of between 19 and 40. See the DMRG section in Methods for more details of the DMRG procedure.

Next, we compile quantum circuits to approximately prepare the MPSs using the AQC-Tensor algorithm^32,33^. AQC-Tensor uses tensor networks to evaluate the fidelity $| \langle \psi | \hat{U}(\vec{\theta })| 0\rangle {| }^{2}$ between a circuit ansatz, $\hat{U}(\vec{\theta })\left\vert 0\right\rangle$, and a target state $\left\vert \psi \right\rangle$, and classically optimises the parameters, $\vec{\theta }$, to maximise the fidelity. To reduce computational overhead, we first compress the ground states whilst maintaining at least 99.9% fidelity, resulting in MPSs with bond dimensions of between 5 and 8, and use these compressed MPSs as the target states for compilation, $\left\vert \psi \right\rangle$. However, we report all fidelities with respect to the uncompressed states. MPS simulation of 1D gapped ground states is known to be computationally efficient^34^, which has been leveraged previously to use AQC-Tensor to prepare ground states of such systems^35^. Due to the exponentially-decaying correlations^36^ present in the ground states considered in this work, we use a brickwork circuit ansatz. Using this method, we obtain circuits which prepare the ${O}_{\frac{1}{2}}$, ${E}_{\frac{1}{2}}$, *E*_−1_, and *E*_−2_ ground states to fidelities of 98.9%, 98.9%, 99.0% and 97.9% with CNOT depths of 18, 21, 21, and 39, respectively. See the Approximate Quantum Compilation section in Methods for more details of the AQC procedure, including the choice of ansatz and the initialisation method. Whilst standard methods for MPS preparation often use staircase circuits^37–41^, we find that these methods were unable to reach comparable fidelities to AQC without prohibitively high circuit depths, for the ground states considered in this work. We refer the reader to Supplementary Information S.I for details of the fidelities and CNOT depths obtained by these methods.

### Quantum hardware experiments

In this section, we present experimental results for the measurement of the string order, edge magnetisation, and the entanglement spectrum, following the procedures sketched in Fig. 2b. See the Experimental Details section in Methods for details of the experimental procedures and error mitigation techniques.

**Fig. 3:**
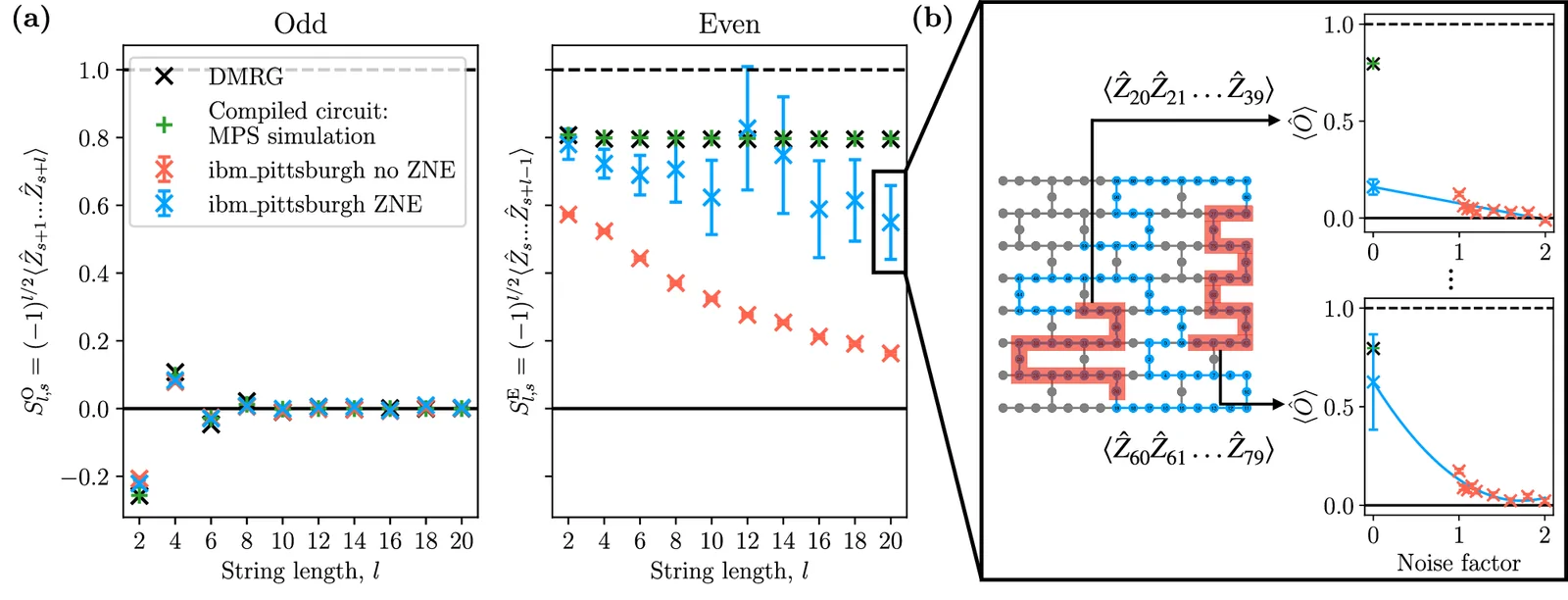
String order parameters of the *E*_−2_ ground state, prepared and measured using ibm_pittsburgh. **a** Odd (left) and even (right) string order parameters as a function of string length. Each data point is averaged over five sections of the chain, corresponding to *s* = 20, 30, 40, 50, and 60. The blue (red) crosses show the expectation values with (without) zero-noise extrapolation (ZNE). The error bars represent the standard error on the mean (red) and the uncertainty of the fit evaluated at a noise factor of zero (blue), after propagation for the average value using the relation for a sum. The black and green crosses show the corresponding values obtained classically using the density matrix renormalisation group (DMRG) algorithm and from matrix product state (MPS) simulation of the compiled circuit, respectively. The black dashed line represents the maximum possible value of 1. **b** A schematic of how the *l* = 20 even string order parameter is obtained by averaging over multiple sections of the chain. (Left) ibm_pittsburgh coupling map showing the qubits used in this experiment (blue) along with the footprints of the 20-local even string order observables (red) for *s* = 20 and 60. (Right) ZNE curves for the two observables. The red crosses show the measured values as a function of noise factor, and the blue curve shows the extrapolation.

Figure 3 a shows the length-*l* string order parameters of Eq. (3), ${S}_{l,s}^{{\rm{E}}/{\rm{O}}}$, with length *l* = 2 to 20 for the *E*_−2_ ground state, as measured on ibm_pittsburgh^42^. Each data point is averaged over five sections of the chain, corresponding to different starting sites for the strings: *s* = 20, 30, 40, 50, and 60. Fig. 3b illustrates the averaging procedure for the *l* = 20 even string order parameter, along with the device footprint for the ${S}_{l = 20,s = 20}^{{\rm{E}}}$ and ${S}_{l = 20,s = 60}^{{\rm{E}}}$ observables. We exclude the first and last 20 sites so that the observables lie within the bulk, minimising edge effects. The red crosses show the expectation values with only the Pauli-twirling^43^ and twirled readout error extinction (TREX)^44^ error mitigation techniques applied, and the blue crosses show the expectation values with additional zero-noise extrapolation (ZNE)^45^. The black and green crosses show the expectation values calculated classically from DMRG and from MPS simulation of the compiled circuit, respectively.

We observe that ${S}_{l,s}^{{\rm{O}}}$ converges towards zero as the string length increases, consistent with the absence of odd string order in the even-Haldane phase. On the other hand, ${S}_{l,s}^{{\rm{E}}}$ remains non-zero even for strings of length *l* = 20, indicating the presence of long-range SPT order. Notably, the expectation values obtained via classical MPS evaluation of the AQC compiled circuit are in excellent agreement with those obtained via DMRG, indicating that the compiled circuit captures the SPT order present in the ground state. In addition to this, the measured data on the hardware without ZNE demonstrates that the SPT order is physically present in the state prepared on the device, over the measured length scale. However, these values monotonically decrease with string length, and do not converge to a constant value. Whether this is due to the compounding effect of measurement readout error on the higher-weight Pauli observables, or an indication that the noise has washed away the order in the state physically realised on the device over greater length scales remains an open question. Results for the other two ground states in the even-Haldane phase, ${E}_{\frac{1}{2}}$ and *E*_−1_, are shown in Supplementary Information S.V. These results are consistent with those of the *E*_−2_ ground state, shown here.

**Fig. 4:**
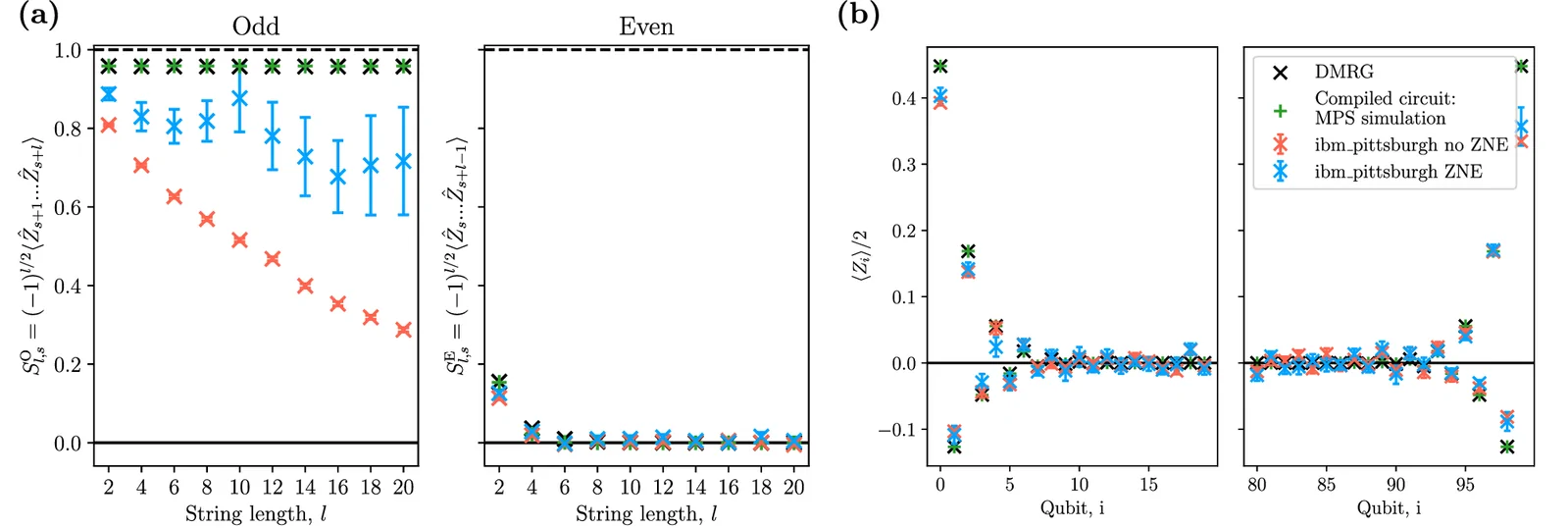
String order parameters and single-site magnetisation of the ${O}_{\frac{1}{2}}$ ground state, prepared and measured using ibm_pittsburgh. **a** Odd (left) and even (right) string order parameters as a function of string length. Each data point is averaged over five sections of the chain,corresponding to *s* = 20, 30, 40, 50, and 60. The black dashed line represents the maximum possible value of 1. **b** Single-site magnetisation, $\langle {\hat{Z}}_{i}\rangle / 2$, of the twenty sites closest to each end of the chain. The fluctuations at the edges correspond to the exponentially-localised spin-1/2 edge states. In both panels, the blue (red) crosses show the expectation values with (without) zero-noise extrapolation (ZNE). The black and green crosses show the corresponding values obtained classically using the density matrix renormalisation group algorithm (DMRG) and from matrix product state (MPS) simulation of the compiled circuit, respectively. The error bars represent the standard error on the mean (red) and the uncertainty of the fit evaluated at a noise factor of zero (blue). In panel (**a**), the uncertainties on the mean over the five sections are calculated using the relation for a sum.

Figure 4 a shows the corresponding ${S}_{l,s}^{{\rm{E}}/{\rm{O}}}$ values for the ${O}_{\frac{1}{2}}$ ground state. The meaning of all elements of the plot is the same as in Fig. 3. In this case we observe complementary behaviour to that in the even-Haldane phase, with ${S}_{l,s}^{{\rm{E}}}$ converging towards zero as the string length increases, and ${S}_{l,s}^{{\rm{O}}}$ remaining non-zero up to length *l* = 20. This is consistent with the presence of odd string order and the absence of even string order in the odd-Haldane phase. The measured string order values allow for an estimation of the fidelity of the state realised on the quantum computer, under an assumed noise model. We refer the interested reader to Supplementary Information S.VI, where we estimate fidelity bounds under the assumption of coherent error or global depolarising noise.

Following on from the string order results, Fig. 4b shows the single-site magnetisation, $\langle {\hat{Z}}_{i}\rangle /2$, of the ${O}_{\frac{1}{2}}$ ground state, for the twenty sites closest to the left end of the chain. These expectation values were measured during the same experiment as the string order, using the same error mitigation techniques, and all elements of the plot have the same meaning. We observe that the expectation values obtained from the hardware agree well with those obtained classically, and exhibit antiferromagnetic ordering close to the edges, with the magnetisation decaying to zero in the bulk.

As discussed previously, the odd-Haldane phase exhibits a symmetry protected, exponentially-localised, spin-1/2 edge state, which we observe as a net magnetisation of 1/2 close to each end of the chain. By grouping lattice sites into two-site unit cells, we fit the magnetisation of each unit cell, ${S}_{1}^{z}=(\langle {\hat{Z}}_{2i}\rangle +\langle {\hat{Z}}_{2i+1}\rangle )/2$, from the left end of the chain, to the functional form: ${S}_{1}^{z}\propto {e}^{-x/{\xi }_{1}}$. From this, we extract a characteristic correlation length, *ξ* = 2*ξ*_1_, of *ξ* = 1.97 ± 0.03 (DMRG and AQC), 1.76 ± 0.20 (hardware, no ZNE), and 1.81 ± 0.21 (hardware, ZNE). The fits are shown in Supplementary Information S.IV. In principle, the correlation length can be used to estimate the energy gap, *Δ*, using the relation *Δ* ~ *v*/*ξ*^46^, provided a suitable estimate for the excitation group velocity, *v*, can be obtained. Here we simply note that the length scale over which the string order is measured is an order of magnitude larger than the correlation length.

**Fig. 5:**
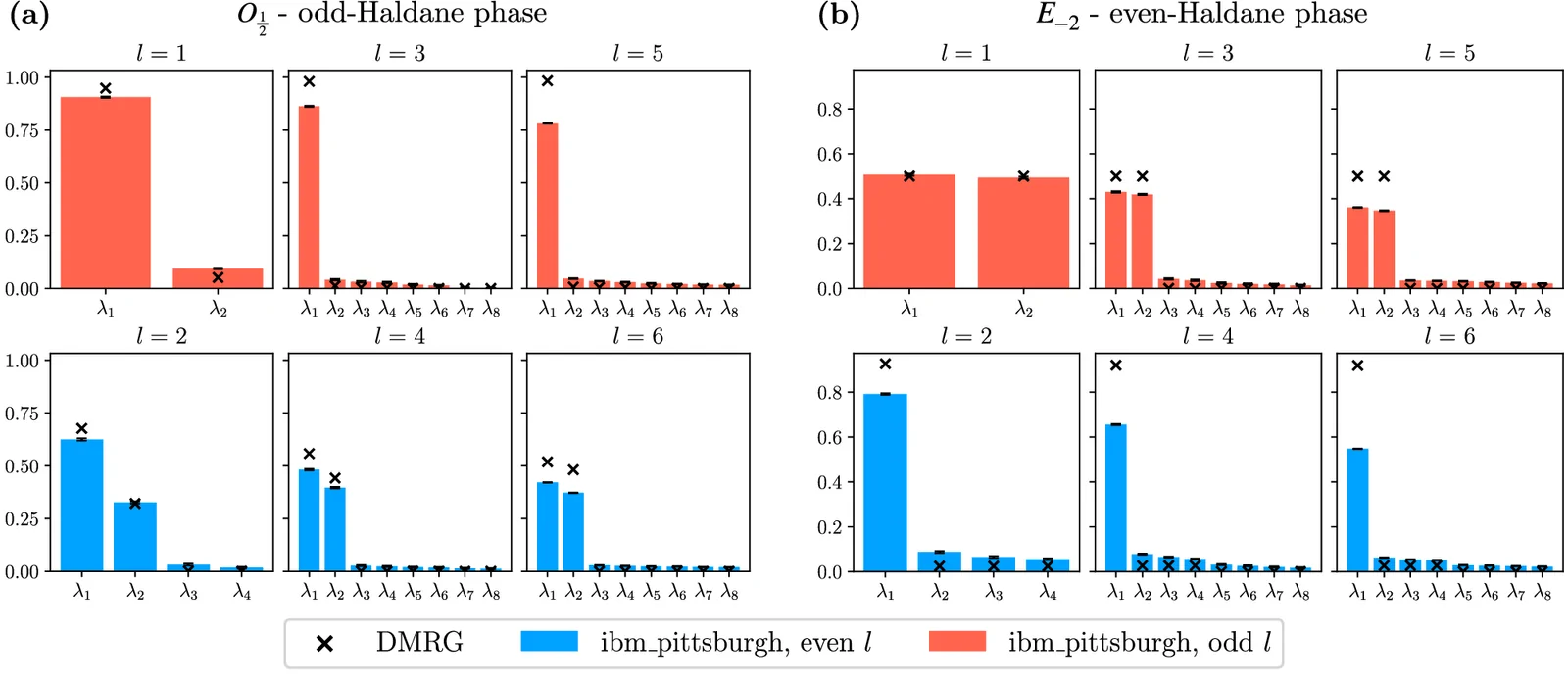
Entanglement spectra in the odd- and even-Haldane phases, measuredusing ibm_pittsburgh. Entanglement spectra of the (**a**) ${O}_{\frac{1}{2}}$ and (**b**) ${E_{-2}}$ ground states for cuts of up to l=6 sites, executed on ibm_pittsburgh. The red (blue) bars show the eigenvalues of the reduced density matrix in descending order, *λ*_1_ ≥ *λ*_2_ ≥…, obtained by cutting *J*_0_ (*J*_1_) bonds. The error bars are obtained via a bootstrapping procedure and represent the standard deviation over 1000 samples of the Pauli-string expectation values. See the Experimental Details section in Methods for more details. The black crosses show the corresponding values obtained classically using the density matrix renormalisation group (DMRG) algorithm.

Finally, we obtain the entanglement spectra of the ${O}_{\frac{1}{2}}$ and *E*_−2_ ground states via state tomography. We construct the reduced density matrices of *l*-site segments from the end of the chain for *l* = 1 to 6, by measuring the expectation values of all *l*-site Pauli strings and combining the values using Eq. (6), as illustrated in Fig. 2b. Our results are shown in Fig. 5. The red (blue) bars represent the eight largest eigenvalues in the spectra obtained by cutting *J*_0_ (*J*_1_) bonds, as measured on ibm_pittsburgh. Here we use the Pauli-twirling and TREX error mitigation techniques, but not ZNE due to the increased overhead arising from the fact that not all of the observables commute. The black error bars in the middle of each bar are obtained using a bootstrapping procedure and show the standard deviation of the values over 1000 samples of the Pauli-string expectation values. The black crosses denote the theoretical expectation, as obtained by DMRG. We do not show the values obtained via MPS simulation of the AQC circuits, since at this scale they are indistinguishable from those from DMRG. See Supplementary Information S.II for a detailed look at the effect of the AQC approximation error.

For the *E*_−2_ ground state (Fig. 5b), we observe an alternating pattern where the entanglement spectra arising from cuts to the *J*_0_ bonds exhibit two dominant eigenvalues, whereas the spectra from cuts to the *J*_1_ bonds exhibit only a single dominant eigenvalue. This observation is qualitatively consistent with the theoretical expectation that the entanglement spectra across *J*_0_ bonds should exhibit even degeneracy, arising from the fact that the entanglement across those bonds cannot be adiabatically removed without crossing a phase boundary, or breaking the symmetry. Whilst we cannot quantitatively argue that the eigenvalues in the spectra are degenerate, we note that this is to be expected, since even a slight error in the reduced density matrix would lead to the eigenvalues not being perfectly degenerate. Results for the other even-Haldane phase ground states, ${E}_{\frac{1}{2}}$ and *E*_−1_, are shown in Supplementary Information S.V and are consistent with the *E*_−2_ results. Looking at the ${O}_{\frac{1}{2}}$ ground state (Fig. 5a), we observe complementary behaviour to the even-Haldane phase, where in this phase, the spectra arising from cuts to the *J*_1_ bonds exhibit two dominant eigenvalues, and those from cuts to *J*_0_ bonds exhibit only a single dominant eigenvalue. In contrast to the even-Haldane phase, in this case the degeneracies of the eigenvalues are only reached with increasing *l*. This is expected due to the influence of the confined edge mode. The suggestion of even degeneracy in the entanglement spectrum across *J*_0_ (*J*_1_) bonds in the even (odd) Haldane phases is a strong indication of the signature symmetry-protection of the entanglement across these bonds.

It should be noted that the even degeneracy of the two eigenvalues in the *l* = 1 spectrum for the *E*_−2_ ground state would also be observed under complete decoherence, where the reduced density matrix of the first site would be described by the maximally mixed state. However, the results from the other spectra indicate that this is not the case, due to the alternating pattern of odd/even degeneracy within each phase, and the complementary patterns observed when comparing the two phases. Both of these observations combine to give strong evidence of the successful preparation of states with SPT order on the quantum hardware.

## Discussion

In this work, we have used an IBM superconducting quantum computer as an experimental platform for realising SPT ground states, as demonstrated with the preparation of 100-site ground states of the BAHC. This was enabled by the combination of DMRG and tensor network AQC, producing high-fidelity (97.9–99.0%), shallow quantum circuits (18–39 CNOT depth). For ground states spanning both the even-Haldane and odd-Haldane SPT phases, we verified the prepared states using multiple characteristic signatures of SPT order. We experimentally observed significant, non-zero string order, qualitative agreement with the expected degeneracies in the entanglement spectrum and, in the odd-Haldane phase, the presence of exponentially-localised, symmetry-protected edge states.

The method presented in this work is well suited to ground states of 1D gapped Hamiltonians with short-range correlations. However, for states with longer-range correlations, it would be important to explore if the required ansatz depth is feasible for current quantum computers. Another direction is to generalise AQC to incorporate mid-circuit measurements and classical feed-forward, which are known to be powerful tools in MPS preparation^47^. Furthermore, understanding how our method can be applied in higher dimensions, where tensor network methods struggle, is of high priority.

Looking forward, an immediate opportunity is the use of digital quantum computers to verify experimental results in SPT physics, such as the recent realisation of the BAHC in nanographene for up to 40 sites^13^. Furthermore, our method could be used to prepare ground states of more complex models, such as those with defects or interfaces between different regions. Importantly, the ground states prepared in this work could be further utilised as an initialisation for the simulation of quench dynamics on quantum computers. In this way, our work represents an important foundation for performing classically challenging many-body simulations of out-of-equilibrium phenomena in SPT systems.

## Methods

### DMRG

For the ground states considered in this work, we obtain their MPS representations using the DMRG algorithm, as implemented in the TeNPy library^31^. For the DMRG procedure, we used at most 20 sweeps (max_sweeps = 20), the default mixer (mixer = True), and combine = True, to reduce computational overhead. When performing the singular value decomposition during DMRG, we keep up to 100 of the largest Schmidt values (chi_max = 100), discard any below 10^−10^ (svd_min = 10^−10^), and discard the smallest values such that the sum of their squares is less than 10^−12^ (trunc_cut = 10^−6^). We refer the reader to the TeNPy documentation for DMRGEngine and truncate for the precise meanings of these configuration options. The bond dimensions and energies of the four ground states considered in this work are shown in the top half of Table 1.

**Table 1:** Bond dimensions, energies, and compiled circuit information for the four ground states considered in this work

State label	${O}_{\frac{1}{2}}$	${E}_{\frac{1}{2}}$	*E*_−1_	*E*_−2_
*J*_0_	0.5	1	1	1
*J*_1_	1	0.5	−1	−2
*χ*	22	19	26	40
*E*	−38.166	−38.819	−41.150	−49.329
*χ* (after compression)	5	5	8	8
*E* (after compression)	−38.164	−38.817	−41.150	−49.329
Compiled fidelity	0.989	0.989	0.990	0.979
Compiled energy	−38.142	−38.794	−41.121	−49.268
Brickwork layers, *L*	3	3.5	3.5	6.5
CNOT depth	18	21	21	39
CNOT count	891	1041	1041	1932

(Top half) Bond dimensions, χ, and energies, *E.* We also report the bond dimensions and energies after compressing to the lowest bond dimension maintaining at least 99.9% fidelity. (Bottom half) Compilation results. We report the compiled circuit with the highest fidelity for each ground state. If multiple circuits reach the same fidelity, we report the one with the lowest depth. Compilation is performed with the compressed states as the targets, but all fidelities are reported with respect to the original, uncompressed states obtained using DMRG.

We note that the ground states in the even-Haldane phase are unique, whereas there is a four-fold degeneracy in the odd-Haldane phase. This is easiest understood by considering the *J*_0_ = 0, *J*_1_ > 0 axis, where the ground state consists of 49 singlet-pairs in the bulk, plus two free spin-1/2s at the edges. In this work, we take the + 1 magnetisation ground state, corresponding to the edge spins being in the *↑↑* configuration.

### Approximate Quantum Compilation

We use AQC to prepare the ground state MPSs as quantum circuits. The goal of AQC is to optimise a variational circuit ansatz to maximise the fidelity with a target state of interest. More precisely, given a target state $\left\vert \psi \right\rangle$, and a variational circuit ansatz $\hat{U}(\vec{\theta })$, AQC seeks to minimise the cost function:

4 $$C=1-| \langle \psi | \hat{U}(\vec{\theta })| 0\rangle {| }^{2}.$$

For the target states, $\left\vert \psi \right\rangle$, we use compressed versions of the MPSs obtained by DMRG (see the DMRG section in Methods), maintaining at least 99.9% fidelity. We perform the compression using the variational method in TeNPy, with between 10 and 50 sweeps. This incurs a small error in the cost function, Eq.(4), compared to using the converged ground state; however, the lower bond dimension reduces the computational cost of evaluating the cost function and its gradients, which we deem to be a worthwhile compromise. Furthermore, the infidelity of the compressed MPSs (≤ 0.1%) is chosen to be an order of magnitude lower than the desired infidelity for AQC (1%). We stress that, whilst we use compressed MPSs as the target states for AQC, we report all fidelities with respect to the original, uncompressed ground states.

To perform AQC, we use the AQC-Tensor add-on for Qiskit^32,33^, which is an efficient implementation, using tensor networks (we use the Quimb package^48^) to evaluate the cost function, Eq. (4), and its gradients.

An important factor for AQC is the choice of ansatz. In this work, we use the *L*-layer nearest-neighbour brickwork ansatz, for its shallow depth and ability to capture short-range correlations. Defining a single brickwork layer as a set of two-qubit unitaries acting on all pairs of qubits (*i*, *i* + 1) with even *i*, followed by an analogous set with odd *i*, the *L*-layer brickwork ansatz is able to exhibit non-zero correlations, ${C}_{zz}(i,\,j)=\langle {\hat{Z}}_{i}{\hat{Z}}_{j}\rangle - \langle {\hat{Z}}_{i} \rangle \langle {\hat{Z}}_{j}\rangle$, for sites separated by up to ∣*i* − *j*∣ = 4*L* − 1. Since the Hamiltonian studied in this work is gapped, its ground states exhibit exponentially decaying correlations^36^, suggesting that they may be well-suited to the brickwork ansatz. It should be noted that the brickwork ansatz may not be well suited for ground states of gapless Hamiltonians, since they often (but not always) exhibit long-range, power-law decaying correlations^36^.

Each two-qubit unitary in the brickwork ansatz is a universal SU(4) unitary. When preparing the circuit ansatz for AQC-Tensor, the following pre-processing steps are applied to the brickwork ansatz: 1. each two-qubit unitary is decomposed using a Cartan decomposition with 15 variational parameters^49^, 2. any single-qubit unitaries are similarly decomposed into a sequence of *R*_*z*_*R*_*x*_*R*_*z*_ gates, 3. the total number of variational parameters is reduced by combining sequences of single-qubit or two-qubit unitaries into corresponding universal SU(4) or SU(2) unitaries, and finally 4. initial *R*_*z*_ rotations acting on the input state, ${\left\vert 0\right\rangle }^{\otimes N}$ are removed.

We choose initial ansatz parameters such that the initial state, $\left\vert {\psi }_{0}\right\rangle =\hat{U}({\vec{\theta }}_{0}){\left\vert 0\right\rangle }^{\otimes N}$, corresponds to the ground state of the model when one of the coupling strengths is zero. This corresponds to a product of singlet states: $\left\vert {\psi }_{{\rm{even}}}\right\rangle ={\left(\left(\left\vert 01\right\rangle -\left\vert 10\right\rangle \right)/\sqrt{2}\right)}^{\otimes N//2}$ for the even-Haldane phase, and a product of singlet states plus two *↑*-configuration edge spins: $\left\vert {\psi }_{{\rm{odd}}}\right\rangle =\left\vert 0\right\rangle \otimes {\left(\left(\left\vert 01\right\rangle -\left\vert 10\right\rangle \right)/\sqrt{2}\right)}^{\otimes N//2-1}\otimes \left\vert 0\right\rangle$ for the odd-Haldane phase. These states can be prepared with a depth-1 circuit, which we append as an extra half brickwork layer to the ansatz for the even-Haldane phase, and incorporate into the final half-layer of the ansatz for the odd-Haldane phase. The fidelities between these initial states and the target ${O}_{\frac{1}{2}}$, ${E}_{\frac{1}{2}}$, *E*_−1_, and *E*_−2_ states are 40%, 46%, 23%, and 2%, respectively, which serve as the starting point for optimisation. Intuitively, the initial fidelity decreases from ${E}_{\frac{1}{2}}$ to *E*_−1_ to *E*_−2_ because these parameterisations are progressively further away from the *J*_1_ = 0 axis in the phase diagram, and so the ground states exhibit less similarity with $\left\vert {\psi }_{{\rm{even}}}\right\rangle$. Finally, we note that due to the singlet-like nature of the ground states, the fidelity with a product state is ${\mathcal{O}}(1{0}^{-10})$ or lower, highlighting the suitability of the singlet initialisation.

The bottom half of Table 1 gives details of the compilation results for the four ground states. We executed multiple compilation runs in parallel, with *L* = 2.5–3.5, 2.5–5.5, and 3.5–6.5 (integer increments) for each of the even-Haldane ground states, respectively. For the ${O}_{\frac{1}{2}}$ ground state, we used *L* = 3, since this was sufficient for the corresponding ${E}_{\frac{1}{2}}$ ground state. Of these parallel runs, we take the one which reached the highest fidelity, and if multiple executions reach the same fidelity, we take the one with the lowest depth. Using the adam optimiser^50^, the AQC procedure was able to reach 98.9%, 98.9%, and 99.0% fidelity for the ${O}_{\frac{1}{2}}$, ${E}_{\frac{1}{2}}$, and *E*_−1_ ground states in a few hours using 3, 3.5, and 3.5 brickwork layers, respectively. In these cases, compilation exited when the target fidelity of 99%, with respect to the compressed target MPS, was reached. For the remaining *E*_−2_ ground state, compilation managed to reach 97.9% fidelity over a 7-day wall-time using 6.5 brickwork layers. We attribute the difficulty of compiling the latter state to the order-of-magnitude lower initial fidelity, and the increased computational overhead of evaluating the cost function and its gradients for the deeper ansatz and higher number of variational parameters.

### Experimental details

For our experimental results, we execute the compiled circuits on quantum hardware and measure the string order, entanglement spectrum, and edge state magnetisation (odd-Haldane phase only). Here we provide details of the experimental setup.

We transpile the compiled circuits to the device using the Qiskit transpiler^51^ using the default settings and optimisation level 3. When choosing a mapping from virtual qubits to physical qubits on the device, the transpiler makes use of the latest calibration data and attempts to avoid particularly error-prone qubits and connections.

The length-*l* string order parameters of Eq. (3), ${S}_{l,s}^{{\rm{E}}/{\rm{O}}}$, correspond to expectation values of Pauli-Z strings of even-length, and left-most index *s* (even for the even parameters and odd for the odd parameters). For example, ${S}_{6,\,20}^{{\rm{E}}}=-\langle {\hat{Z}}_{20}{\hat{Z}}_{21}{\hat{Z}}_{22}{\hat{Z}}_{23}{\hat{Z}}_{24}{\hat{Z}}_{25}\rangle$. The string order parameters, Eq. (2), correspond to the *l* → *∞* limit.

We measure the length-*l* string order parameters for *l* = 2, 4, . . . , 20 over five sections of the chain corresponding to *s* = 20, 30, 40, 50, and 60, avoiding 20 sites from either end of the chain to avoid edge effects. We execute the AQC compiled circuits on the ibm_pittsburgh quantum computer and measure the expectation values using the Qiskit^51^ Estimator primitive. For each observable, we use 10 000 shots, TREX^44^, Pauli-twirling^43^ with 100 randomisations, and ZNE^45^. For the ZNE procedure, we use the noise factors: 1, 1.05, 1.1, 1.15, 1.2, 1.4, 1.6, 1.8, and 2.0, and we use the best-fit extrapolator, from the choice of exponential, quadratic, and linear, with the following two exceptions:

1. If the range of the noisy data is below 0.1, we use a linear extrapolation.
2. If the extrapolated value is unphysical (greater than 1 or less than −1 for Pauli strings), we discard this and choose the next-best fit.

We determine the best fit as that with the lowest Bayesian information criterion^52^, which is a heuristic designed to favour models with a low sum of squared residuals (SSR), whilst penalising models with more parameters (to avoid over-fitting) and is given by:

5 $${\rm{BIC}}=n\,{\ln }({\rm{SSR}}/n)+k\,{\ln }(n),$$

where *n* is the number of data points and *k* is the number of parameters in the extrapolation function.

After obtaining the extrapolated values of ${S}_{l,s}^{{\rm{E}}}$ and ${S}_{l,s}^{{\rm{O}}}$, we average the values for each *l* over the values of *s*, and propagate the corresponding uncertainties using the relation for a sum. We note that we use the same experimental setup when measuring the single-qubit magnetisations $\langle {\hat{Z}}_{i}\rangle$, but in that case we do not average over multiple windows.

Our results for the *E*_−2_ and ${O}_{\frac{1}{2}}$ ground state experiments are shown in Fig. 3 and Fig. 4, and used 1m 35s and 1m 29s of QPU time, respectively.

Immediately before our circuit is executed on the quantum device, we execute a circuit with the same two-qubit gate structure, but the action of which, in the absence of noise, corresponds to the identity, and perform the ZNE procedure on individual Pauli-Z observables. With the knowledge that $\langle \hat{{Z}_{i}}\rangle =1$ in the absence of noise, we can validate our choice of noise factors, and check for any particularly error-prone qubits. This is similar to the calibration procedure discussed in ref. ^53^. For more details on this validation technique, we refer the reader to Supplementary Information S.III.

We use tomography to measure the reduced density matrices of *l*-site segments, using the equation:

6 $${\hat{\rho }}^{(l)}=\frac{1}{{2}^{l}}\sum _{{k}_{1},\,...,\,{k}_{l}}\langle {\hat{\sigma }}_{{k}_{1}}...{\hat{\sigma }}_{{k}_{l}}\rangle {\hat{\sigma }}_{{k}_{1}}...{\hat{\sigma }}_{{k}_{l}},$$

with each ${\hat{\sigma }}_{{k}_{i}}\in \{\hat{I},\,\hat{X},\,\hat{Y},\,\hat{Z}\}$^54^. The number of required observables scales as 4^*l*^ which, even after grouping into commuting groups (206 as opposed to 4096 for *l* = 6), incurs a significant resource overhead to measure. For this reason, we do not use ZNE for this experiment. Whilst more efficient methods exist for performing state tomography^55^, we use this method for simplicity.

Using the Qiskit^51^Estimator primitive, we measure the expectation values of all length-*l* Pauli strings on the ibm_pittsburgh device. For each observable, we use 10 000 shots, TREX^44^, and Pauli-twirling^43^ with 100 randomisations. It should be noted that we do not need to repeat the experiment for all values of *l*, since all Pauli strings of length *a* < *l* are contained in the set of Pauli strings of length *l*.

From the measured expectation values, we construct the reduced density matrix, ${\hat{\rho }}^{(l)}$, and compute its eigenvalues {*λ*_*i*_}, with *λ*_1_ ≥ *λ*_2_ ≥ . . . . To estimate the uncertainties of the eigenvalues, we use a bootstrapping procedure. We generate *k* = 1 000 samples of ${\hat{\rho }}^{(l)}$, denoted as ${\hat{\rho }}_{j}^{(l)}$ for *j* ∈ {1, . . . , *k*} by sampling *k* values of each Pauli string expectation value $\langle {\hat{\sigma }}_{{k}_{1}}...{\hat{\sigma }}_{{k}_{l}}\rangle$, from a normal distribution with the same mean and standard deviation as measured from the quantum device. Then we transform the sampled matrix ${\hat{\rho }}_{j}^{(l)}$ into the eigenbasis of the average matrix ${\hat{\rho }}^{(l)}$, and take the diagonal entries $\{{\lambda }_{i}^{j}\}$. We then take the mean and standard deviation of each value, ${\lambda }_{i}^{j}$ over the *k* samples (*j* = 1, . . . , *k*).

Our results for the ${O}_{\frac{1}{2}}$ and *E*_−2_ ground state experiments, using *l* = 6, are shown in Fig. 5a and b, requiring 44m 46s and 48m 18s of QPU time, respectively.

## Supplementary information


Supplementary Information


## Data Availability

The data that support the findings of this study are available at https://github.com/stfc/spt-order.

## Code availability

The code that supports the findings of this study is available at https://github.com/stfc/spt-order.
