# Supplementary material for: Symmetry-protected topological order in a 100-site spin chain on a digital quantum computer
Source: npj Quantum Inf. 2026 Sep 1;12(1):141. doi: 10.1038/s41534-026-01334-8 (PMC13533844; doi:10.1038/s41534-026-01334-8)
Supplement: Supplementary file 1 [file 41534_2026_1334_MOESM1_ESM.pdf]

Supplementary Information

This supplementary information is structured as follows. In Sec. S.I we compare the fidelities and CNOT depths achieved by two commonly-used matrix product state (MPS) preparation techniques to those achieved by approximate quantum compiling (AQC). In Sec. S.II we look at the effect of the infidelity introduced by AQC on the entanglement spectra. In Sec. S.III we detail a technique used to verify that the zero-noise extrapolation (ZNE) procedure gives sensible results for circuits with known noiseless expectation values. In Sec. S.IV we show fits for the magnetisation at the edges of the chain, used to extract the correlation length. In Sec. S.V we give further experimental results for the ground states not shown in the main text. Finally, in Sec. S.VI we estimate bounds on the experimental fidelity of the states realised on the quantum device.

S.I. COMPARISON TO OTHER MPS PREPARATION TECHNIQUES

Two commonly used mehods for preparing MPSs as quantum circuits make use of circuits with a staircase, or ladder, structure. The first of these methods is the only exact method of MPS preparation, first introduced in [S1], and explicitly detailed in [S2]. After gauge transforming the MPS into a certain canonical form, each tensor is embedded in a unitary, introducing $|0\rangle$-initialised ancilla qubits as necessary. This results in a staircase of unitaries, where each unitary requires $1 + \lceil \log_2 \chi \rceil$ qubits, where χ is the local bond dimension. Whilst exact, this method often results in circuits with high circuit depth, due to the complexity of decomposing the large unitaries into single and two-qubit gates. We refer to this method as the "exact ladder (EL)" method.

The second method, related to the EL method, is an approximate method explicitly detailed in [S3], with related methods proposed in [S1, S4]. The method involves iteratively compressing an MPS to bond dimension $\chi = 2$, preparing the compressed MPS exactly as a ladder of two-qubit unitaries using the EL method, applying the inverse of the resulting circuit to the non-compressed MPS, and repeating. The circuit after L-iterations of this procedure consist of L ladder layers of two-qubit unitaries which approximately prepares the target state. We refer to this method as the "approximate ladder (AL)" method.

Table S1 compares the fidelity and circuit depths achieved by these methods, using the open source implementation [S5], to those achieved by AQC. For a given ground state, we apply the methods to two compressed versions of the state, one with at least 99.9% fidelity, and the other with at least 98% fidelity. The first compressed target state (top half of Table S1) is the same state as was used as the target for AQC and the second compressed target state (bottom half of Table S1) is intended to allow the other methods a similar infidelity as was obtained by AQC.

State label	$O_{\frac{1}{2}}$			$E_{\frac{1}{2}}$			E_{-1}			E_{-2}		
Target χ	5			5			8			8		
Target fidelity	0.999			0.999			1.000			1.000		
	Fidelity	CNOT depth	CNOT count	Fidelity	CNOT depth	CNOT count	Fidelity	CNOT depth	CNOT count	Fidelity	CNOT depth	CNOT count
AQC	0.989	18	891	0.989	21	1041	0.990	21	1041	0.979	39	1932
EL	0.999	24,069	24,639	0.999	20,096	21,066	1.000	20,145	20,715	1.000	20,246	20,816
AL ($L = 1$)	0.607	297	297	0.597	297	297	0.376	297	297	0.078	297	297
AL ($L = 10$)	0.803	351	2970	0.798	351	2970	0.644	351	2970	0.343	351	2970
Target χ	4			4			4			7		
Target fidelity	0.999			0.999			0.994			0.989		
	Fidelity	CNOT depth	CNOT count	Fidelity	CNOT depth	CNOT count	Fidelity	CNOT depth	CNOT count	Fidelity	CNOT depth	CNOT count
EL	0.999	3368	3368	0.999	3383	3383	0.994	3379	3379	0.989	20,496	21,066
AL ($L = 1$)	0.607	297	297	0.597	297	297	0.376	297	297	0.078	297	297
AL ($L = 10$)	0.806	351	2970	0.801	351	2970	0.648	351	2970	0.317	351	2970

TABLE S1. Comparison of the AQC, EL, and AL compilation techniques. (Top) fidelities, CNOT depths, and CNOT counts of the circuits produced by the three techniques, when using the same target MPS as was used for AQC. Here L refers to the number of ladder layers used for the AL method. (Bottom) results for the EL and AL methods when used with a target MPS compressed to at least 98% fidelity. All fidelities are calculated with respect to the original, uncompressed ground state MPS obtained by DMRG.

We note that all fidelities in Table S1 are calculated with respect to the original, uncompressed ground state MPS obtained by DMRG.

Over the four ground states, the AL method only manages to reach fidelities of between 0.078 and 0.806, whilst producing circuits of CNOT depth between 297 and 351. This is compared to fidelities of 0.979 to 0.990 with CNOT depths between 18 and 39 for the AQC method, highlighting the suitability of the brickwork ansatz for these ground states. The EL method manages to reach very high fidelities of 0.979 to 1.000, at the expense of extremely deep circuits, with CNOT depths between 3368 and 24,069: well beyond the capabilities of current generation quantum devices.

We note that other methods for MPS preparation exist, such as ones using a renormalisation-group-based ansatz [S6, S7] or adaptive circuits with mid-circuit measurement and classical feedforward [S8], but we do not expect these approaches to perform favourably for the states under study. The former requires a circuit depth scaling quartically with bond dimension, while the latter is non-deterministic when strict symmetry requirements are not obeyed. We therefore do not conduct an exhaustive comparison with all possible methods for MPS preparation, and narrow our focus to AQC due to its favourable balance of fidelity and circuit depth.

S.II. THE EFFECT OF AQC APPROXIMATION ON THE ENTANGLEMENT SPECTRUM

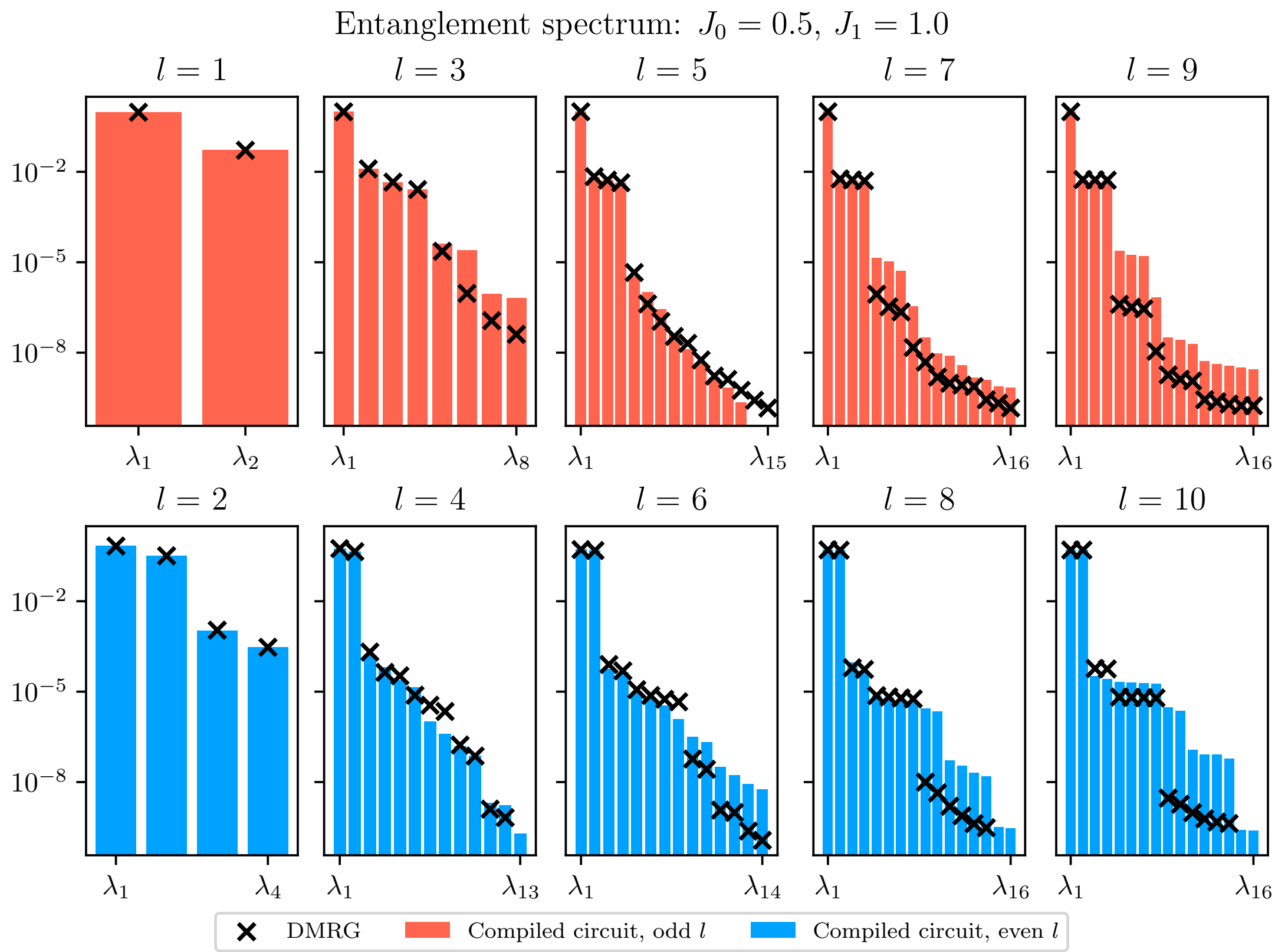

FIG. S1. The effect of AQC approximation error on the entanglement spectrum of the $O_{\frac{1}{2}}$ ground state. The red (blue) bars show up to the sixteen largest eigenvalues, λ_i, of the l-site reduced density matrix obtained by cutting J_0 (J_1) bonds, calculated via MPS simulation of the AQC compiled circuit. The black crosses show the corresponding values obtained from DMRG. We discard any values below 10^{-10}.

In the experiments section of the main text, we present experimental results for the entanglement spectrum of the $O_{\frac{1}{2}}$ and E_{-2} ground states obtained using `ibm_pittsburgh` (Fig. 5 in the main text). We compare the results to the corresponding spectrum obtained by DMRG. However, we do not show the entanglement spectrum obtained by MPS simulation of the AQC compiled circuit, since is is almost indistinguishable from the DMRG spectrum on the scale of the plot. In this section, we look at the effect of the AQC approximation error on the entanglement spectrum.

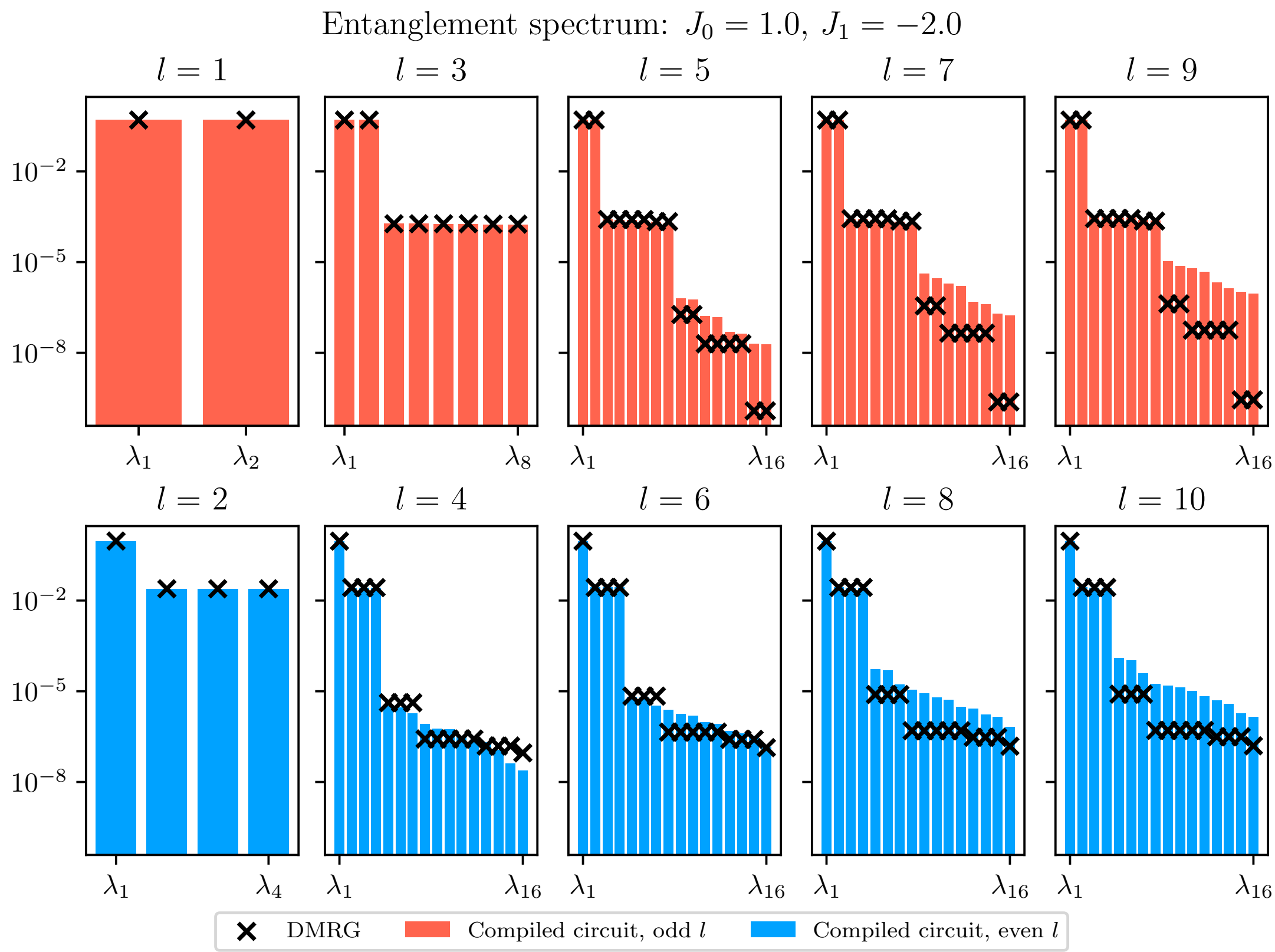

FIG. S2. The effect of AQC approximation error on the entanglement spectrum of the E_{-2} ground state. The red (blue) bars show up to the sixteen largest eigenvalues, λ_i, of the l-site reduced density matrix obtained by cutting J_0 (J_1) bonds, calculated via MPS simulation of the AQC compiled circuit. The black crosses show the corresponding values obtained from DMRG. We discard any values below 10^{-10}.

Fig. S1 and Fig. S2 show the entanglement spectrum for cuts of length $l = 1$ to 10 for the $O_{\frac{1}{2}}$ and E_{-2} ground states respectively. The red and blue bars show the values obtained via classical MPS simulation of the AQC compiled circuit, and the black crosses show those obtained from DMRG. We observe that the entanglement spectrum values from DMRG and from the AQC compiled circuit are in good agreement down to a scale of around 10^{-4}. Below 10^{-4}, the values are not captured well by AQC, and the signature symmetry-protected topological (SPT) degeneracies break down.

S.III. IDENTITY CIRCUIT ZNE

An important consideration when using the ZNE error mitigation technique [S9] is the choice of noise factors. If the chosen noise factors cover a range that is too small, there would be very little variation in the measured expectation value, and fitting procedures would be unstable. On the other hand, if the range of noise factors is too large, the measured expectation values would quickly lose any information from the circuit. Following the idea of using circuits with known noiseless expectation values for calibrating the noise factors mentioned in [S10], we use a technique using so called "identity circuits" to verify that the chosen range of noise factors used is suitable.

Our AQC circuits consist of a brickwork structure of layers of SU(4) universal two-qubit unitaries, decomposed using the Cartan decomposition [S11]. The parameters of each SU(4) unitary are determined by the AQC procedure. We can construct a circuit with the same structure as the AQC circuit, but which corresponds to the identity operation by choosing parameters such that each SU(4) unitary corresponds to the identity. When transpiling the circuit to the device with the Qiskit [S12] transpiler, using optimisation level 1 ensures that the transpiler does not remove the identity SU(4) unitaries. With such a circuit, in the absence of noise, the expectation value of single-qubit Pauli-Z operators is: $\langle \hat{Z}_i \rangle = 1$. Ideally, performing the ZNE procedure with such a circuit, we expect a) the unmitigated (noise factor 1) value should be close to 1 b) this value should decay fairly monotonically and smoothly over the

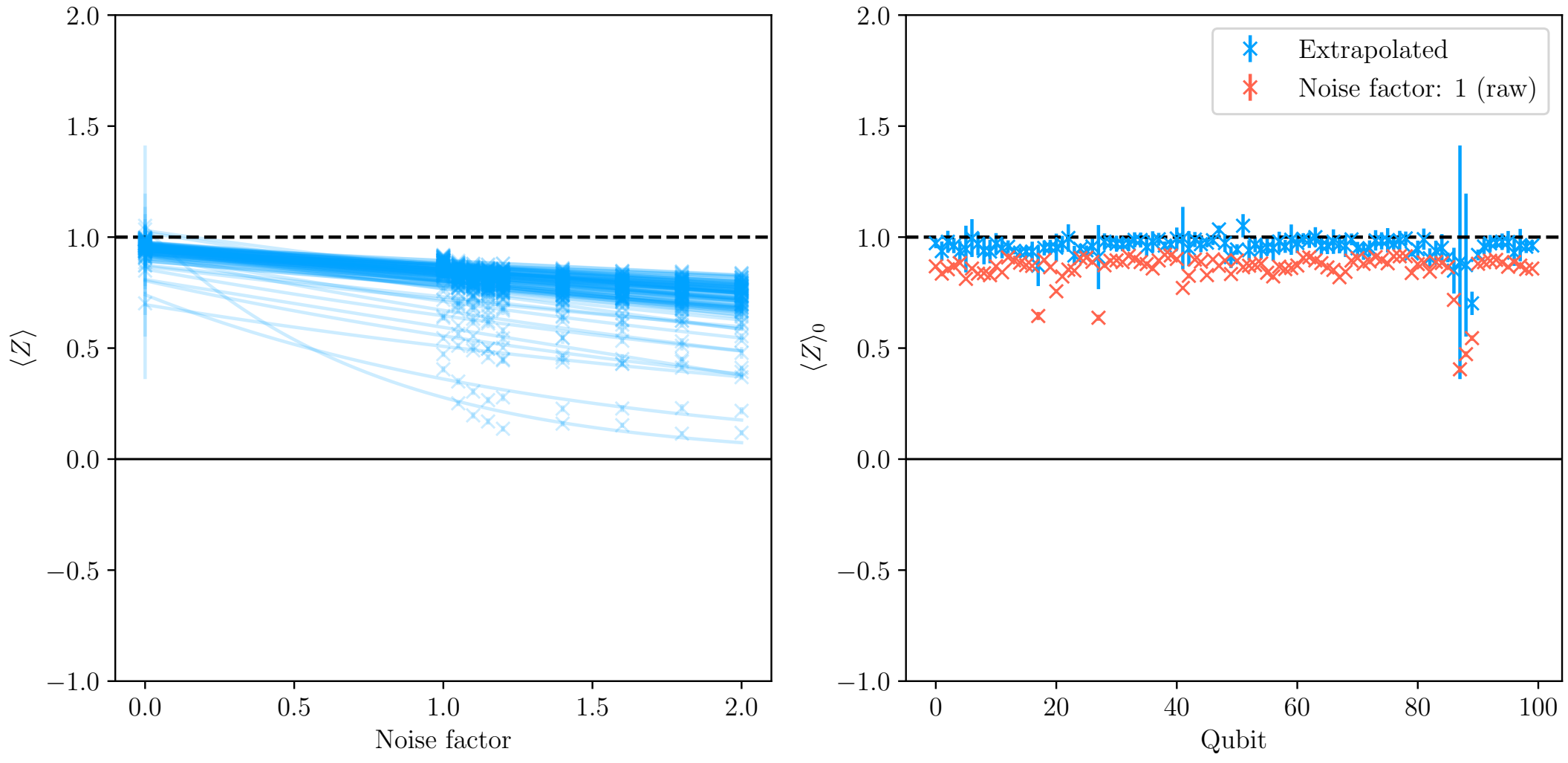

FIG. S3. Results of the identity ZNE procedure for the experiment shown in Fig. 3, using **ibm_pittsburgh**. (Left) Pauli-Z expectation value, $\langle \hat{Z}_i \rangle$ as a function of noise factor, for all qubits ($i = 0, \ldots, 99$). (Right) Extrapolated value (blue, noise factor 0) and unmitigated value (red, noise factor 1) as a function of virtual qubit index.

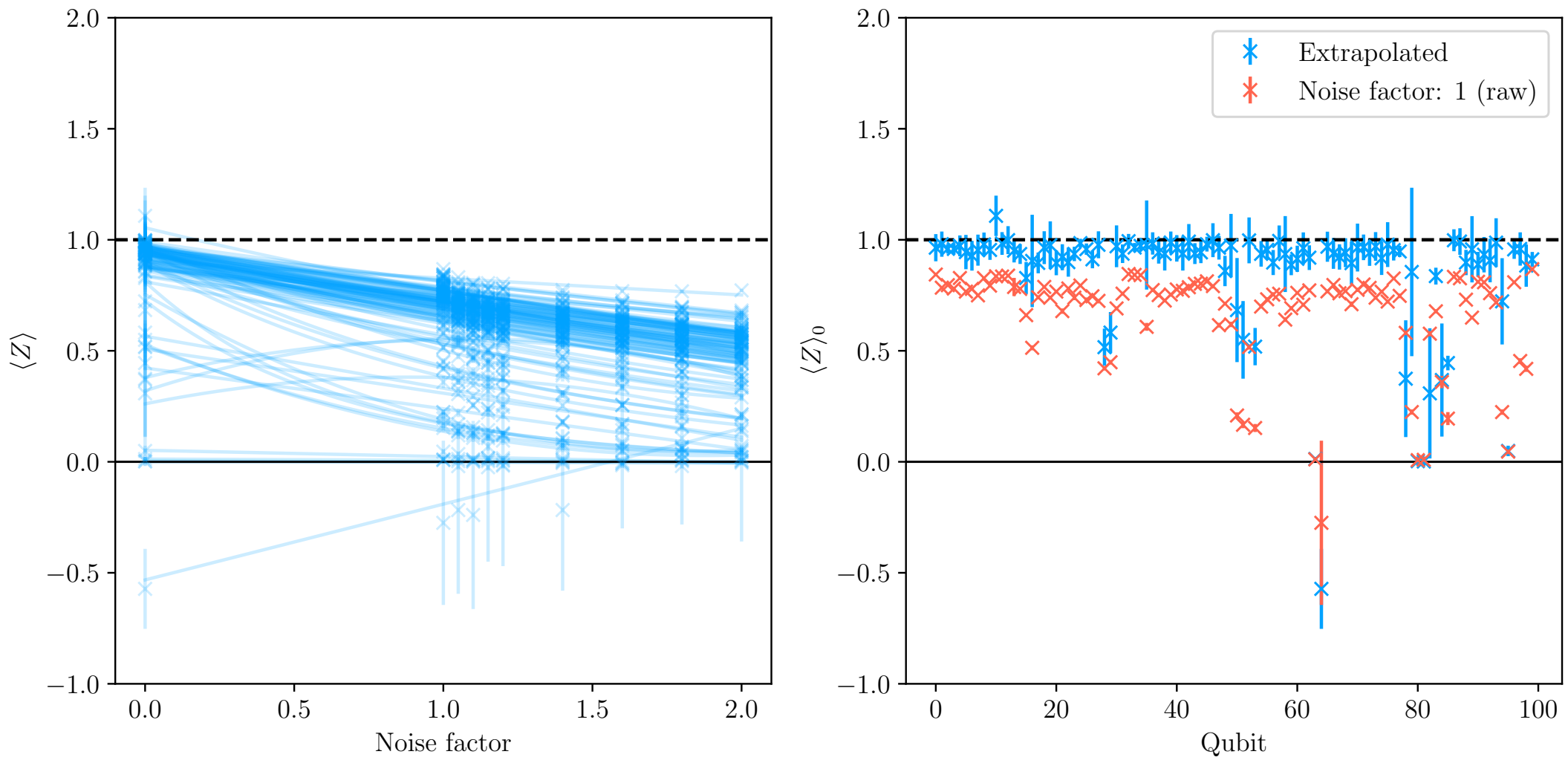

FIG. S4. Example of the effect of bad qubits on the identity ZNE procedure, using **ibm_fez**. (Left) Pauli-Z expectation value, $\langle \hat{Z}_i \rangle$ as a function of noise factor, for all qubits ($i = 0, \ldots, 99$). (Right) Extrapolated value (blue, noise factor 0) and unmitigated value (red, noise factor 1) as a function of virtual qubit index.

range of noise factors, and c) when extrapolated to zero noise, the value should be close to 1. If we observe that the unmitigated value is close to zero, or the values behave erratically, then the circuit is likely too deep for the device, or the range of noise factors is too large. Another possibility is that the chosen circuit layout on the quantum device could include one or more particularly noisy qubits. In this case, such a qubit should stand out when performing ZNE with the identity circuit.

Immediately before executing the AQC circuits in our experiments, we execute an identity circuit as described above, and perform the ZNE procedure. We use the same circuit layout on the quantum device as is used for the real experiment. Fig. S3 shows the results of such an experiment, performed on **ibm_pittsburgh** immediately prior to the experiment shown in Fig. 3 in the main text, using an identity circuit with the same structure and layout as the E_{-2} compiled circuit. We observe that the unmitigated $\langle \hat{Z} \rangle$ expectation values mostly lie between 0.8 and 0.9, with the lowest value around 0.4. The behaviour of the expectation values with increasing noise factor is smooth, with no

erratic jumps, and the extrapolated values all lie close to the true value of 1. There are also no qubits which behave significantly differently from the rest. We conclude that the ZNE procedure with the identity circuit is working as intended, and we can be confident that: the circuit is not too deep for the device, the range of noise factors is suitable, and the transpiler did not choose a layout with any particularly noisy qubits. From this, we infer that the same holds for the real experiment with the compiled circuit.

Fig. S4 shows the effect of bad qubits on the identity ZNE procedure, using `ibm_fez`. Here, multiple qubits exhibit expectation values which are either all close to zero, or oscillate erratically as a function of noise factor, and result in poor extrapolations. Qubit 64 appears to be particularly bad, with a negative extrapolated expectation value. In this case, we conclude that the chosen circuit layout included particularly error-prone qubits, and discard the experiment.

S.IV. EDGE MAGNETISATION DECAY

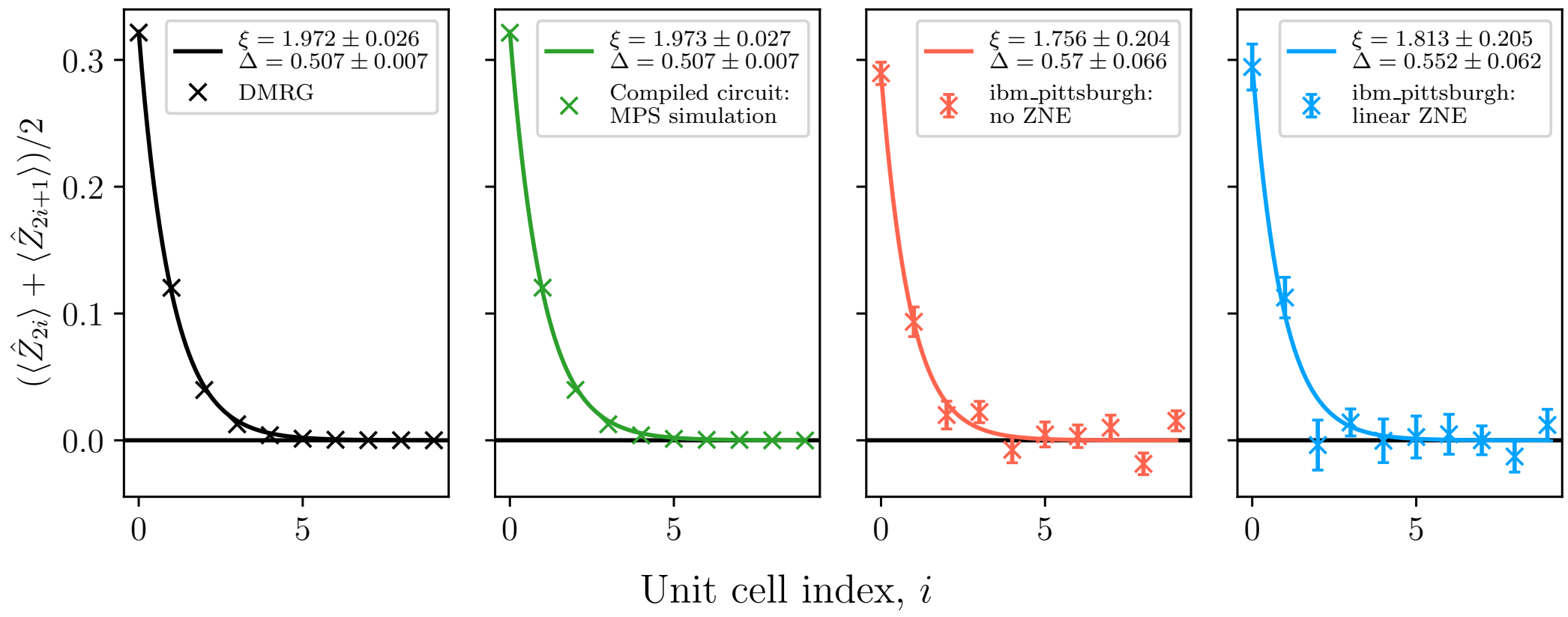

FIG. S5. Fits of the magnetisation of each two-site unit cell, $S_1^z = (\langle \hat{Z}_{2i} \rangle + \langle \hat{Z}_{2i+1} \rangle)/2$, from the left end of the chain, to the functional form: $S_1^z \propto e^{-x/\xi_1}$. The blue (red) crosses show the expectation values obtained using `ibm_pittsburgh` with (without) ZNE. The black and green crosses show the corresponding values obtained classically from DMRG and from MPS simulation of the compiled circuit, respectively.

In Fig. 4b in the main text we show the single-site magnetisation for the twenty sites closest to each end of the chain, for the $O_{\frac{1}{2}}$ ground state, obtained using `ibm_pittsburgh`. As detailed in the results discussion, we fit the magnetisation of each two-site unit cell, $S_1^z = (\langle \hat{Z}_{2i} \rangle + \langle \hat{Z}_{2i+1} \rangle)/2$, from the left end of the chain, to the functional form: $S_1^z \propto e^{-x/\xi_1}$, and use this to extract the correlation length, $\xi = 2\xi_1$. Fig.S5 shows the results of the fitting procedure. We obtain $\xi = 1.97 \pm 0.03$ (DMRG and AQC), 1.76 ± 0.20 (hardware, no ZNE), and 1.81 ± 0.21 (hardware, ZNE).

S.V. FURTHER EXPERIMENTAL RESULTS

Fig. 3 and Fig. 4 in the experiments section of the main text shows the string order parameters as a function of string length, for the E_{-2} and $O_{\frac{1}{2}}$ ground states respectively, as measured using `ibm_pittsburgh`. Here, Figures S6 and S7 show the string order parameters, $S^{\mathrm{O}}_{l,s}$ (left) and $S^{\mathrm{E}}_{l,s}$ (right), for the $E_{\frac{1}{2}}$, and E_{-1} ground states, respectively. The blue and red crosses show the expectation values as measured using `ibm_pittsburgh` with and without using ZNE, respectively. The black and green crosses show the corresponding quantities obtained classically using DMRG and MPS simulation of the compiled circuits, respectively. The values in the plot are averaged over five sections of the chain, corresponding to $s = 20$, 30, 40, 50, and 60. The results are consistent with those of the E_{-2} ground state: $S^{\mathrm{O}}_{l,s}$ decays to zero, consistent with the absence of odd string order, and $S^{\mathrm{E}}_{l,s}$ remains non-zero even up to $l = 20$, indicating the presence of even string order.

Fig. 5 in the main text shows the entanglement spectra of the $O_{\frac{1}{2}}$ and E_{-2} ground states, obtained using `ibm_pittsburgh`, for cuts of length $l = 1$ to 6. Here, Figures S8 and S9 show the entanglement spectra of the $E_{\frac{1}{2}}$ and E_{-1} ground states, respectively, for cuts of length $l = 1$ to 4. The red (blue) bars show the eight largest

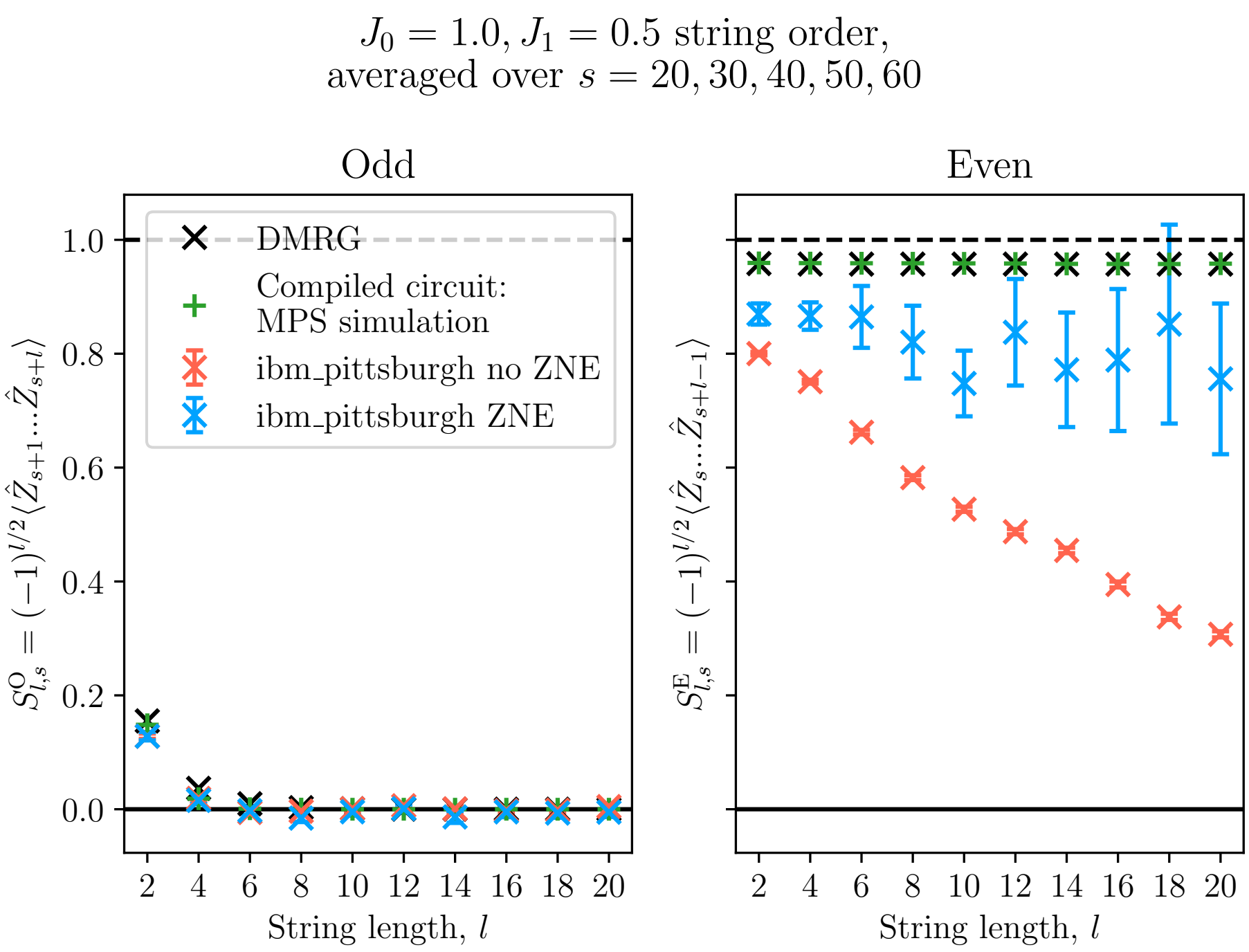

FIG. S6. String order parameters for the $E_{\frac{1}{2}}$ ground state, averaged over five sections of the chain, obtained using `ibm_pittsburgh`. The blue and red crosses show the expectation values as measured using `ibm_pittsburgh` with and without using ZNE, respectively. The error bars represent the standard error on the mean (red) and the uncertainty of the fit evaluated at a noise factor of zero (blue), after propagation for the average value using the relation for a sum. The black and green crosses show the corresponding quantities obtained classically using DMRG and MPS simulation of the compiled circuits, respectively. The black dashed line represents the maximum possible value of 1.

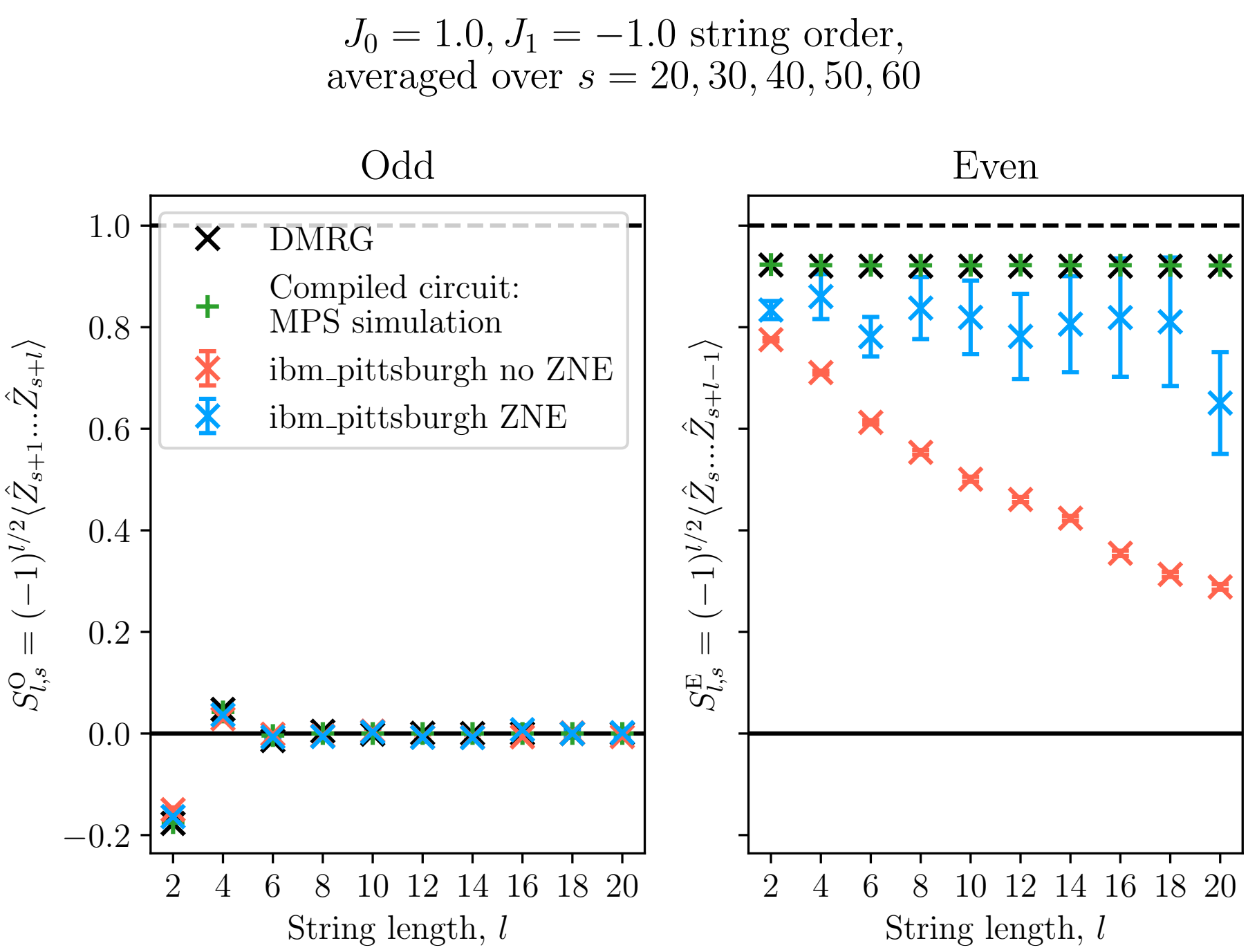

FIG. S7. String order parameters for the E_{-1} ground state, averaged over five sections of the chain, obtained using `ibm_pittsburgh`. The meanings of all elements of the plot are the same as in Fig. S6.

eigenvalues, $\lambda_1 \geq \ ... \ \geq \lambda_8$, of the reduced density matrix obtained by cutting J_0 (J_1) bonds. The black crosses show the corresponding values obtained from DMRG. The results are consistent with those of the E_{-2}, with spectra resulting from cuts to J_0 bonds having two dominant eigenvalues, and those from cuts to J_1 bonds having only a single dominant eigenvalue.

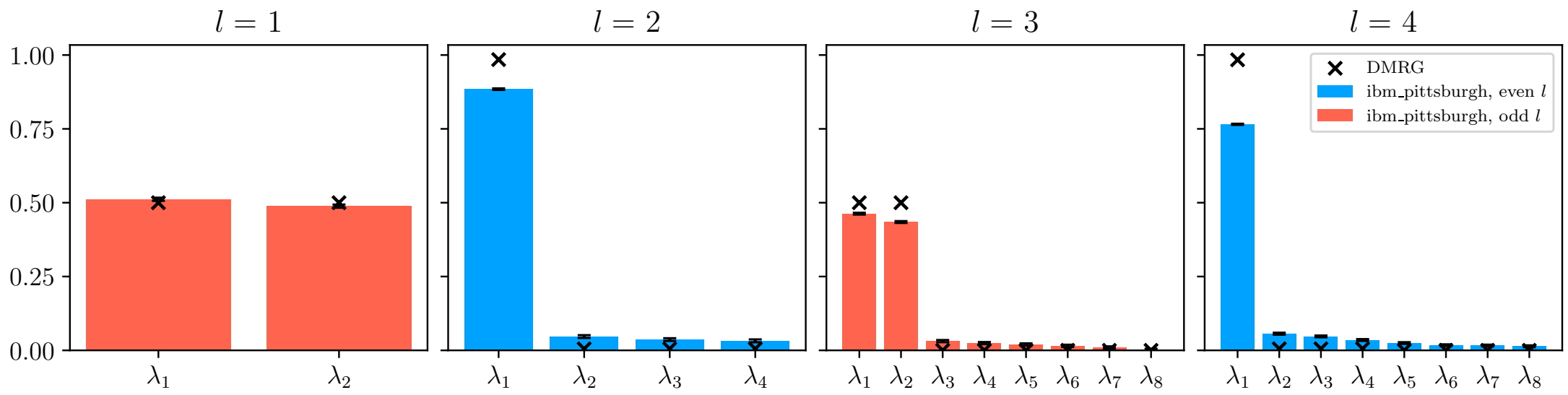

FIG. S8. Entanglement spectra of the $E_{\frac{1}{2}}$ ground state for cuts of up to $l = 4$ sites, executed on `ibm_pittsburgh`. The red (blue) bars show the eight largest eigenvalues, $\lambda_1 \geq \ ... \ \geq \lambda_8$, of the reduced density matrix obtained by cutting J_0 (J_1) bonds. The black crosses show the corresponding values obtained from DMRG. The error bars are obtained via a bootstrapping procedure, the Experimental Details section in Methods for more details.

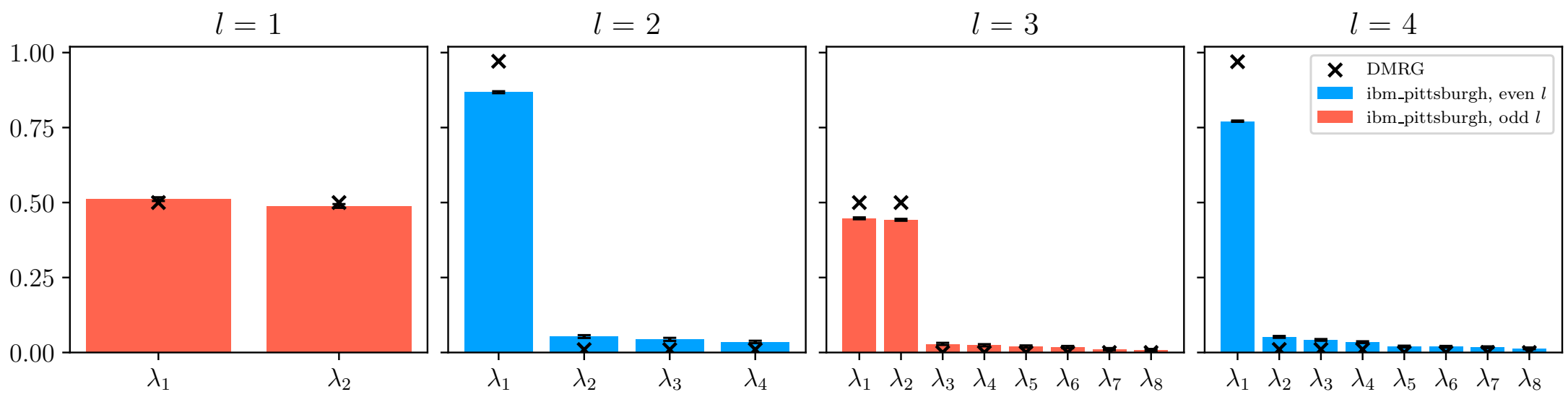

FIG. S9. Entanglement spectra of the E_{-1} ground state for cuts of up to $l = 4$ sites, executed on `ibm_pittsburgh`. The meanings of all elements of the plot are the same as in Fig. S8.

S.VI. NOISY FIDELITY ESTIMATES

In this section, we present theoretical estimates for the fidelity of the states physically prepared on the quantum hardware, using the measured values of the string order parameter. We consider two simple models: coherent error, and a global depolarising channel.

A. Coherent error

In this case, upon executing the AQC compiled circuits on the quantum device, the state, $|\psi\rangle$, remains pure and may be written as:

$$|\psi\rangle = \sqrt{F}\,|\psi_0\rangle + \sqrt{1-F}\,|\psi_\perp\rangle\,, \tag{S1}$$

where F is the fidelity, $|\psi_0\rangle$ is the ideal state, and $|\psi_\perp\rangle$ is a state in the subspace orthogonal to $|\psi_0\rangle$. From this, we have that:

$$\langle\psi|\hat{O}|\psi\rangle = F\,\langle\psi_0|\hat{O}|\psi_0\rangle + 2\sqrt{F(1-F)}\mathrm{Re}\,\langle\psi_0|\hat{O}|\psi_\perp\rangle + (1-F)\,\langle\psi_\perp|\hat{O}|\psi_\perp\rangle\,. \tag{S2}$$

Using the general relations for expectation values and matrix elements of operators: $E_{\rm min} \leq \langle\psi|\hat{O}|\psi\rangle \leq E_{\rm max}$ and $|{\rm Re}\,\langle\psi|\hat{O}|\phi\rangle\,| \leq E_{\rm max}$, where $E_{\rm min}$ and $E_{\rm max}$ are the minimum and maximum eigenvalues of $\hat{O}$, and the fact that $E_{\rm min} = -1$ and $E_{\rm max} = 1$ for the string order parameter, we can bound ${\rm Re}\,\langle\psi_0|\hat{O}|\psi_\perp\rangle$ and $\langle\psi_\perp|\hat{O}|\psi_\perp\rangle$ to lie between -1 and 1. With this, we have two inequalities:

$$\langle\psi|\hat{O}|\psi\rangle \leq F\,\langle\psi_0|\hat{O}|\psi_0\rangle + 2\sqrt{F(1-F)} + 1 - F \tag{S3}$$

$$\langle\psi|\hat{O}|\psi\rangle \geq F\,\langle\psi_0|\hat{O}|\psi_0\rangle - 2\sqrt{F(1-F)} - 1 + F. \tag{S4}$$

Numerically solving the first of these inequalities for our data always gives the bound $0 \leq F \leq 1$, which isn't particularly useful. The second inequality is more interesting. As a worst-case estimate, we can consider taking $\langle\psi|\hat{O}|\psi\rangle$ to either be the lowest measured string order parameter (even or odd in the even- and odd-Haldane phases respectively) or zero, since the measured string order parameters appear to tend to zero for larger strings. This gives the following bounds:

State	$\langle\psi_0\|\hat{O}\|\psi_0\rangle$	$\langle\psi\|\hat{O}\|\psi\rangle$	Bound
$O_{\frac{1}{2}}$	0.9573	0.2874	$0 \leq F \leq 0.926$
		0	$0 \leq F \leq 0.863$
$E_{\frac{1}{2}}$	0.9573	0.3072	$0 \leq F \leq 0.929$
		0	$0 \leq F \leq 0.863$
E_{-1}	0.9201	0.2887	$0 \leq F \leq 0.932$
		0	$0 \leq F \leq 0.871$
E_{-2}	0.7949	0.1630	$0 \leq F \leq 0.931$
		0	$0 \leq F \leq 0.897$

This implies that the states realised on the device have lower fidelity than in the noiseless case, which is not surprising, but does not provide a non-zero lower bound.

B. Global depolarising channel

Assuming a global depolarising channel, the density matrix describing the state prepared on the quantum device, ρ, is given by:

$$\rho = (1-\lambda)\rho_0 + \frac{1}{2^N}\mathbb{1}, \tag{S5}$$

where $\rho_0 = |\psi_0\rangle\langle\psi_0|$ is the ideal pure state. The fidelity is $F = \left({\rm tr}\sqrt{\sqrt{\rho_0}\rho\sqrt{\rho_0}}\right)^2 = {\rm tr}(\rho\rho_0) = (1-\lambda)+\lambda/2^N \approx (1-\lambda)$, where we have used the fact that the ideal state is pure. Under this channel, the string order expectation values become:

$$\begin{aligned} {\rm tr}(\rho\hat{O}) &= (1-\lambda){\rm tr}(\rho_0\hat{O}) + \frac{\lambda}{2^N}{\rm tr}(\hat{O}) && \text{(S6)}\\ &= (1-\lambda)\,\langle\psi_0|\hat{O}|\psi_0\rangle && \text{(S7)}\\ &\approx F\,\langle\psi_0|\hat{O}|\psi_0\rangle\,, && \text{(S8)} \end{aligned}$$

where we have used the fact that $\frac{\lambda}{2^N}{\rm tr}(\hat{O}) = 0$ both because Pauli observables are traceless, and N is large. From this, we can estimate the fidelity from the ratio of the measured and ideal string order parameters:

$$F = \frac{{\rm tr}(\rho\hat{O})}{\langle\psi_0|\hat{O}|\psi_0\rangle}. \tag{S9}$$

Using the lowest measured (non-zero) string order parameter would yield fidelities of 0.300, 0.321, 0.314, and 0.205 for the $O_{\frac{1}{2}}$, $E_{\frac{1}{2}}$, E_{-1}, and E_{-2} states, respectively, and using a projected value of 0 string order at large l would yield a fidelity of 0. However, clearly a global depolarising channel does not accurately describe the device noise, since the ratio between the measured and ideal string order parameters varies with the string length. This would imply that the noise is more localised.

C. Conclusion

Under the assumption of either coherent errors or a global depolarising channel, loose theoretical bounds on the fidelity of the state prepared on the hardware may be obtained. However, neither assumption is particularly representative of the actual noise channel on the device, and the fidelity bounds are unlikely to be accurate.

[S1] C. Schön, E. Solano, F. Verstraete, J. I. Cirac, and M. M. Wolf, Sequential generation of entangled multiqubit states, Physical review letters **95**, 110503 (2005).
[S2] S.-H. Lin, R. Dilip, A. G. Green, A. Smith, and F. Pollmann, Real- and imaginary-time evolution with compressed quantum circuits, PRX Quantum **2**, 010342 (2021).
[S3] S.-J. Ran, Encoding of matrix product states into quantum circuits of one-and two-qubit gates, Physical Review A **101**, 032310 (2020).
[S4] C. Schön, K. Hammerer, M. M. Wolf, J. I. Cirac, and E. Solano, Sequential generation of matrix-product states in cavity qed, Phys. Rev. A **75**, 032311 (2007).
[S5] D. A. Millar, G. W. Pennington, N. T. M. Siow, and S. J. Thomson, qiskit-community/mps-to-circuit (2025).
[S6] M. Scheer, A. Baiardi, E. B. Marty, Z.-Y. Wei, and D. Malz, Renormalization-group-based preparation of matrix product states on up to 80 qubits, arXiv preprint arXiv:2510.24681 (2025).
[S7] D. Malz, G. Styliaris, Z.-Y. Wei, and J. I. Cirac, Preparation of matrix product states with log-depth quantum circuits, Phys. Rev. Lett. **132**, 040404 (2024).
[S8] K. C. Smith, A. Khan, B. K. Clark, S. Girvin, and T.-C. Wei, Constant-depth preparation of matrix product states with adaptive quantum circuits, PRX Quantum **5**, 030344 (2024).
[S9] Z. Cai, Multi-exponential error extrapolation and combining error mitigation techniques for nisq applications, npj Quantum Information **7**, 80 (2021).
[S10] R. Majumdar, P. Rivero, F. Metz, A. Hasan, and D. S. Wang, Best practices for quantum error mitigation with digital zero-noise extrapolation (2023), arXiv:2307.05203 [quant-ph].
[S11] F. Vatan and C. Williams, Optimal quantum circuits for general two-qubit gates, Phys. Rev. A **69**, 032315 (2004).
[S12] A. Javadi-Abhari, M. Treinish, K. Krsulich, C. J. Wood, J. Lishman, J. Gacon, S. Martiel, P. D. Nation, L. S. Bishop, A. W. Cross, B. R. Johnson, and J. M. Gambetta, Quantum computing with Qiskit (2024), arXiv:2405.08810 [quant-ph].
